# Involvement of CTCF in transcription regulation of EGR1 at early G1 phase as an architecture factor

**DOI:** 10.1038/s41598-018-36753-x

**Published:** 2019-01-23

**Authors:** Takeshi Sekiya, Kohsuke Kato, Atsushi Kawaguchi, Kyosuke Nagata

**Affiliations:** 1 Department of Infection Biology, Graduate School of Comprehensive Human Science, University of Tsukuba, Tsukuba, Japan; 2 Department of Infection Biology, Faculty of Medicine, University of Tsukuba, Tsukuba, Japan

## Abstract

Early growth response 1 (EGR1) is a transcription factor and regulates cellular processes such as proliferation, differentiation, and apoptosis. The expression of EGR1 is rapidly induced in response to several stimuli, and it activates the expression of downstream target genes involved in signaling cascades. *EGR1* gene is also known to be transcribed in early G1 phase. However, the regulation of *EGR1* transcription in early G1 phase is not clarified well. Here we found that CCCTC-binding factor (CTCF), a chromatin binding protein, is required to transcribe *EGR1* gene at the onset of early G1 phase. We found that CTCF mediated the formation of higher-order chromatin structures among CTCF binding sites located in the *EGR1* locus. Disruption of the CTCF-dependent higher-order chromatin structure using nuclease-dead Cas9 (dCas9)-mediated interference reduced the *EGR1* transcription in early G1 phase. Collectively, we propose that CTCF has functional roles for the temporal expression of *EGR1* in early G1 phase through regulation of higher-order chromatin structure organization.

## Introduction

Immediate early genes (IEGs) are a set of cellular genes, and transcription of mRNAs from which is rapidly induced by both extracellular and intracellular signals through several factors which does not requires *de novo* protein synthesis^1^. Most IEGs encode transcription factors involved in initiation of signaling cascades by modulating transcription of the target genes. *EGR1* belongs to IEGs and is transcribed rapidly and transiently in response to various kinds of stimuli^2^. EGR1 functions as both an activator and a repressor for transcriptional regulation of numerous genes, including *EGF*, *TGF-β*, and *Bax*, in concert with several cofactors^3,4^ to control cell growth, tumorigenesis^5^, and apoptosis^6^. Analyses of the *EGR1* promoter revealed that a number of *cis-*elements are located within 720 bp upstream of the *EGR1* transcription start site (TSS)^7^. It has been proposed that several serum response elements (SREs) located at approximately 300 bp upstream of the *EGR1* TSS have a crucial role for the expression of *EGR1*^7^. Serum response factor (SRF) binds to SREs by interacting with TCF co-regulator proteins in a phosphorylation-dependent manner^8^. It has been reported that the expression of *EGR1* was induced in early G1 phase^9^. Because SRF-TCF complex is activated in early G1 phase by growth factors to induce genes involved in G1 progression^10^, *EGR1* is thought to be regulated by SRF-TCF complex in early G1 phase. From functional analyses of CTCF in the *EGR1* expression, CTCF was thought to function as a negative regulator during mouse myeloid cell differentiation or in LPS-stimulated macrophages^11^. CTCF binding motif is located at approximately 1.2 kb upstream of the *EGR1* TSS^11^. CTCF is a DNA binding protein possessing C2H2 zinc finger motifs and was originally found as a repressor of *c-myc*^12^ and *lysozyme*^13^. CTCF is a highly conserved multifunctional protein in vertebrates. CTCF functions not only as a transcription factor with a variety of binding partners^14^, but also a regulator of higher-order chromatin structure through the formation of chromatin loops that define the boundary between transcriptionally active and inactive chromatin^15^. Recently we reported that CTCF is highly phospholylated in mitosis and the phosphorylation of CTCF impairs its DNA binding activity^16^. In parallel with the reformation of nucleus at telophase, dephosphorylation of CTCF and its recruitment on chromatin might occur, although a phosphatase(s) involved in this process is Unknown. Higher order chromatin structures, including structures possibly regulated by CTCF, are disrupted in mitosis and reformed in G1 phase^17^. Regulation of gene expressions through higher-order chromatin structures are observed through whole cell cycles. In particular, early G1 phase may be a time in which profound reformation of higher-order chromatin structure and transcription restart simultaneously proceed. Thus, CTCF might be involved in the expression of early G1 genes, such as *EGR1*. However, the precise function of CTCF in the *EGR1* expression in early G1 phase is not well known.

Here, we have shown that CTCF is required for the transcription of *EGR1* in early G1 phase. Chromatin Immunoprecipitation (ChIP) and Chromosome Conformation Capture (3 C) analyses indicated that CTCF-mediated higher-order chromatin structure is formed among the promoter and the upstream and the downstream CTCF-binding sites of the *EGR1* gene locus after mitotic exit. dCas9-mediated interference of the formation of higher-order chromatin structure in early G1 phase also reduced *EGR1* transcription. Collectively, these results suggest that CTCF is important for the temporal transcription regulation of *EGR1* through its function in the organization of higher-order chromatin structure.

## Results

### CTCF is required for the expression of the *EGR1* gene in early G1 phase

**Figure 1:**
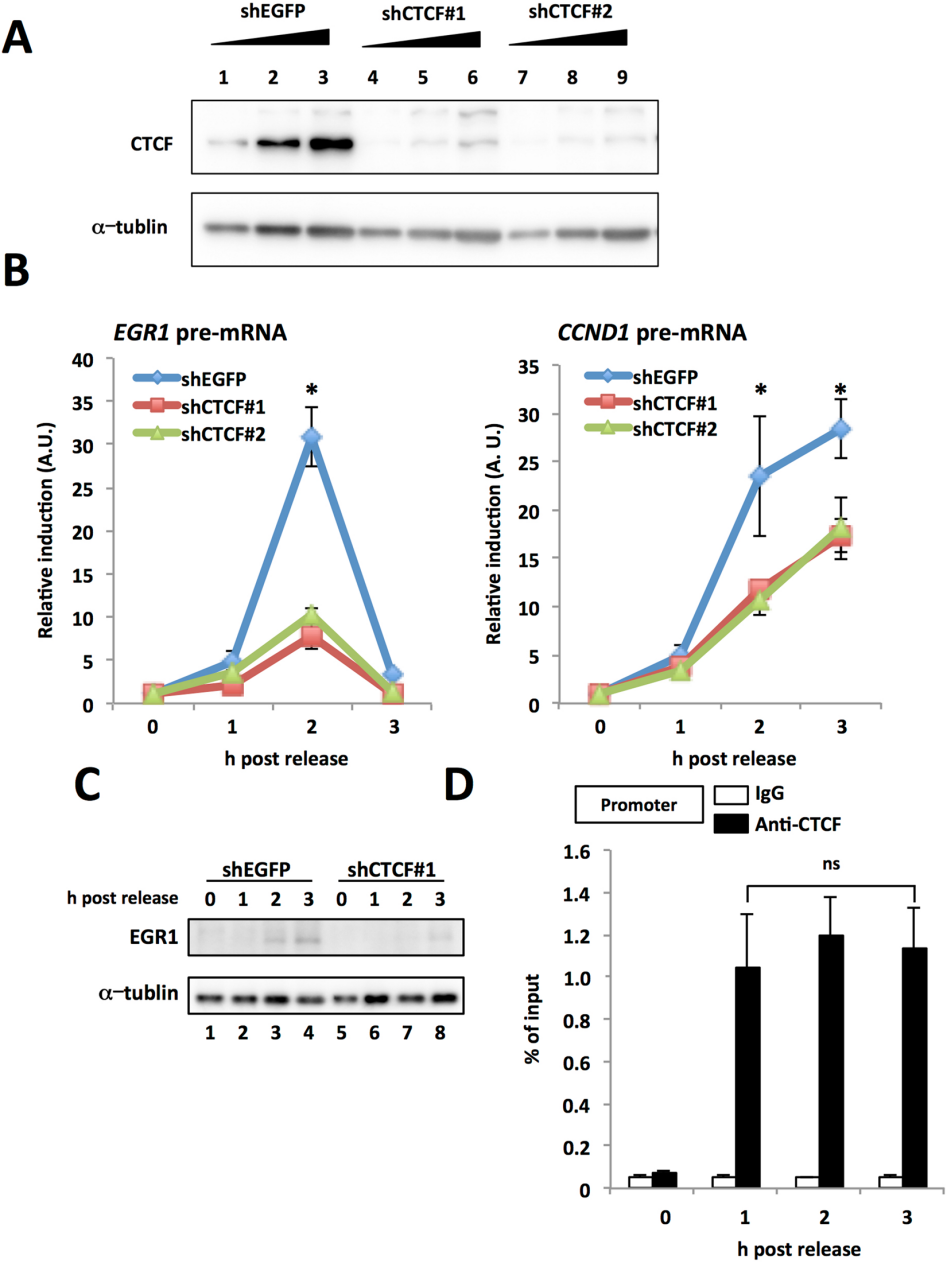
CTCF was associated with *EGR1* promoter and stimulated its transcription in early G1 phase. (**A**) Expression level of CTCF in CTCF KD cells. HeLa S3 cells were transfected with shEGFP expression plasmid as a control (shEGFP, lanes 1–3) or shCTCF#1 expression plasmid (shCTCF#1, lanes 4–6), or shCTCF#2 expression plasmid (shCTCF#2, lanes 7–9). Lysates from desired cell numbers (0.25 × 10^5^, 0.5 × 10^5^, and 1 × 10^5^) were subjected to western blot analyses using anti-CTCF and anti-α-tublin antibodies. The original blots are presented in Supplementary Fig. S9. (**B**) Effect of CTCF KD on transcription in early G1 phase. HeLa S3 cells transfected with shEGFP (blue line and diamonds) or shCTCF#1 (red line and squares), or shCTCF#2 (green line and triangles) expression plasmid were synchronozied and arrested with nocodazole. Total RNAs were extracted from cells collected at indicated time points after nocodazole release and subjected to qRT-PCR using specific primers for *EGR1* pre-mRNA, *CCND1* pre-mRNA, and 28 S *rRNA*. The amounts of pre-mRNA levels were normalized by the amount of 28 S *rRNA*, and then relative expression levels were shown. (**C**) Expression level of EGR1 in CTCF KD cells in early G1 phase. HeLa S3 cells were transfected with shEGFP expression plasmid (shEGFP, lanes 1–4) or shCTCF#1 expression plasmid (shCTCF#1, lanes 5–8). Lysates from cells collected at indicated time points were subjected to western blot analyses using anti-EGR1 and anti-α-tublin antibodies. The original blots are presented in Supplementary Fig. S9. (**D**) ChIP analysis for quantification of the level of CTCF associated with the promoter-proximal CTCF binding site in early G1 pahse. Eary G1 cells were collected at indicated time points after release, and ChIP assays were performed using with anti-CTCF antibody and rabbit normal IgG. qPCR was performed with primers for amplification of DNA including CTCF binding sites located the 1.2 kb upstream of the *EGR1* gene transcription start site (TSS). The relative amount of DNA co-immunoprecipitated with each antibody was shown as % of input. Data information: Each error bar shows standard deviation of three independent experiments. Student’s two tailed t-test, *P < 0.05, ns, *P* > 0.05.

To know whether CTCF is involved in the expression of *EGR1* in early G1 phase, we examined the effect of CTCF knockdown (KD) on the *EGR1* transcription level in early G1 phase. In CTCF KD cells, using plasmids expressing shRNA against CTCF (shCTCF#1 and #2), the expression level of the CTCF proteins was less than 25% of that in the control cells (Fig. 1A). At 63 h post transfection of the shRNA expression plasmid, HeLa S3 cells were treated with 165 nM of nocodazole for 6 h, as described in the Experimental procedures. The expression levels of CTCF were not affected by cell cycle synchronization (Supplementary Fig. S1). After removal of the drug, the cells were incubated at 37 °C to synchronize the cell population in early G1 phase. Total RNAs were isolated from the cells and subjected to qRT-PCR using the primers that span the exon-intron junctions. Along with the progression of G1 phase, the expression level of *EGR1* pre-mRNA was peaked at 2 h post release and quickly reduced at 3 h post release in the control cells (Fig. 1B). In contrast, the *EGR1* transcription level in CTCF KD cells had decreased to less than 30% of that in the control cells at 2 h post release (Fig. 1B). These results indicate that CTCF is a positive regulator of *EGR1* transcription in early G1 phase. We also examined the pre-mRNA level of *CCND1* gene which is also expressed in G1 phase and has putative CTCF binding sites^18^. The amount of *CCND1* pre-mRNA was reduced in CTCF KD cells compared with that of control cells, suggesting that CTCF also regulates *CCND1* transcription in G1 phase. Similar results were obtained from shCTCF#1 and shCTCF#2. The cell cycle progression profiles of the control and CTCF KD cells were not significantly changed (Supplementary Fig. S2). Notably, the expression of EGR1 protein also reduced in CTCF KD cells in early G1 phase (Fig. 1C). To clarify the role(s) of CTCF in the transcriptional regulation of *EGR1*, we examined the binding of CTCF to a CTCF binding site located 1.2 kb upstream^11^ of the *EGR1* TSS during early G1 phase. ChIP assays were performed using lysates prepared from HeLa S3 cells at 0, 1, 2 and 3 h post release from nocodazole treatment. As expected, CTCF interacted with the CTCF binding site in the *EGR1* promoter after nocodazole release and its binding was observed during cell cycle progression (Fig. 1D).

### CTCF does not affect early G1 transcription from the *EGR1* promoter in a reporter plasmid system

**Figure 2:**
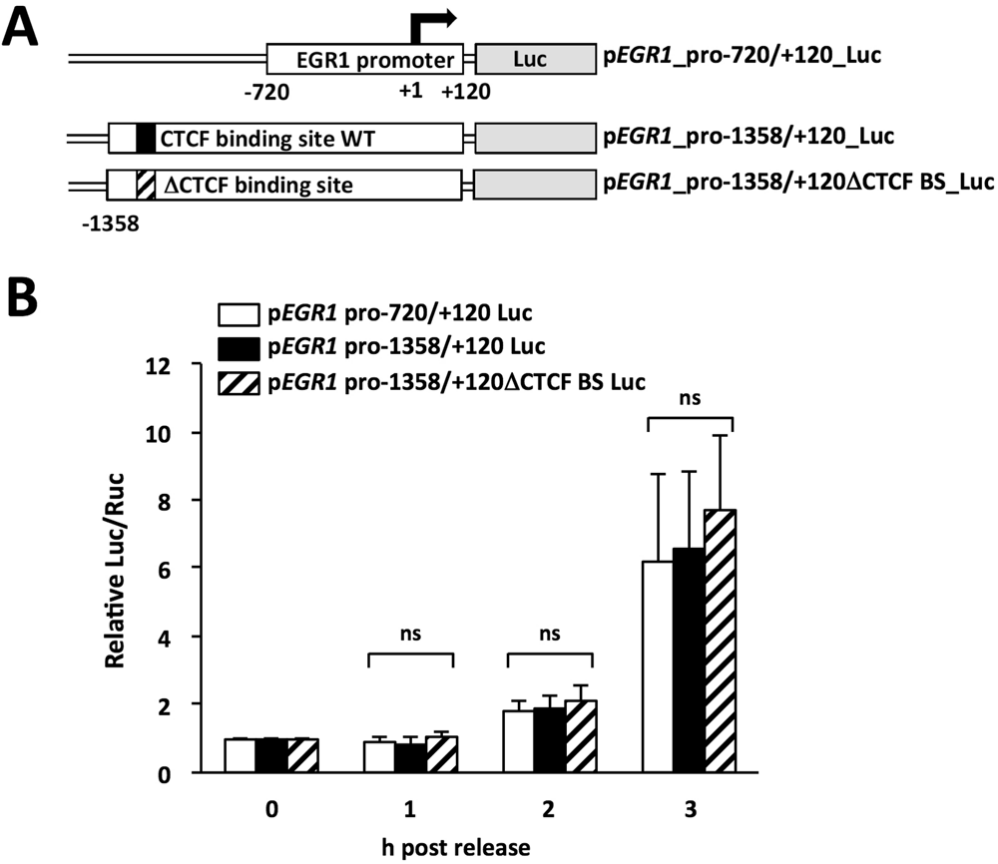
CTCF did not affect early G1 transcription from the *EGR1* promoter in reporter plasmid-transfected cells. (**A**) Schematic diagram of human *EGR1* promoter luciferase reporter plasmids. (**B**) The CTCF binding site (BS) located at 1.2 kb upstream of TSS is not required for temporal transcription regulation of *EGR1* in early G1 phase. HeLa S3 cells were transfected with p*EGR1* pro-720/+120 Luc, p*EGR1* pro-1358/+120 Luc, p*EGR1* pro-1358/+120ΔCTCF BS Luc plasmids together with pRL-SV40 as a normalizer. Cells were synchronized and arrested by nocodazole. Eary G1 cells were collected at indicated time points after release. The firefly luciferase activity was normalized with the renilla luciferase activity and represented as relative values. Data information: Each error bar shows standard deviation of three independent experiments. Student’s two tailed t-test, ns, *P* > 0.05.

**Figure 3:**
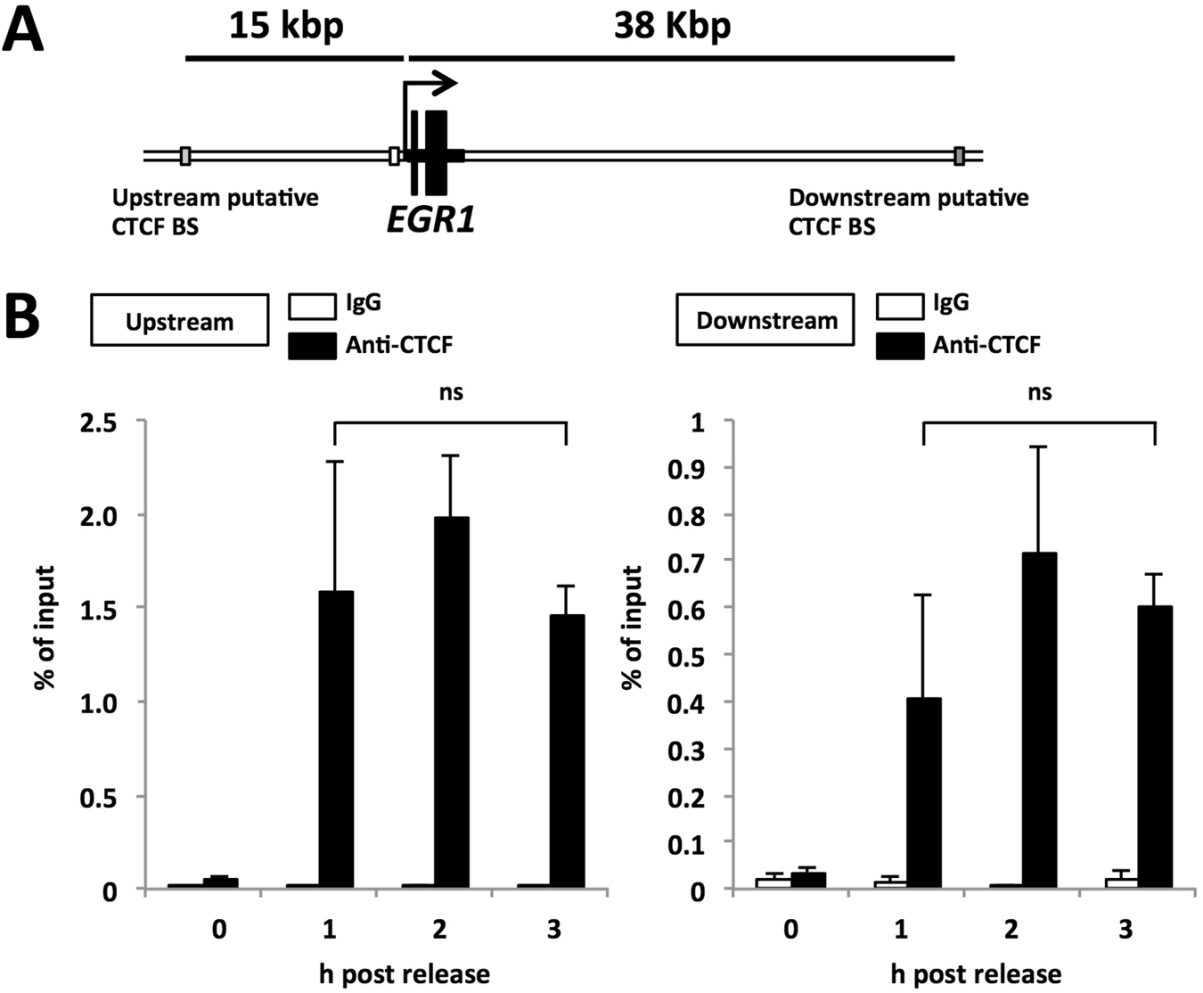
CTCF bound upstream and downstream CTCF binding sites in early G1 phase. (**A**) Schematic diagram of the human *EGR1* gene locus and CTCF binding sites. (**B**) ChIP analysis for quantification of the CTCF level associated with the distal CTCF binding sites. The lysates were prepared as described in the legend for Fig. 1D and subjected to immunoprecipitation with anti-CTCF antibody and rabbit normal IgG. qPCR was performed with primer sets for amplification of the upstream or downstream CTCF bindig sites of the *EGR1* gene. The amount of DNA co-immunoprecipitated with each antibody was shown as % of input. Data information: Each error bar shows standard deviation of three independent experiments. Student’s two tailed t-test, ns, *P* > 0.05.

**Figure 4:**
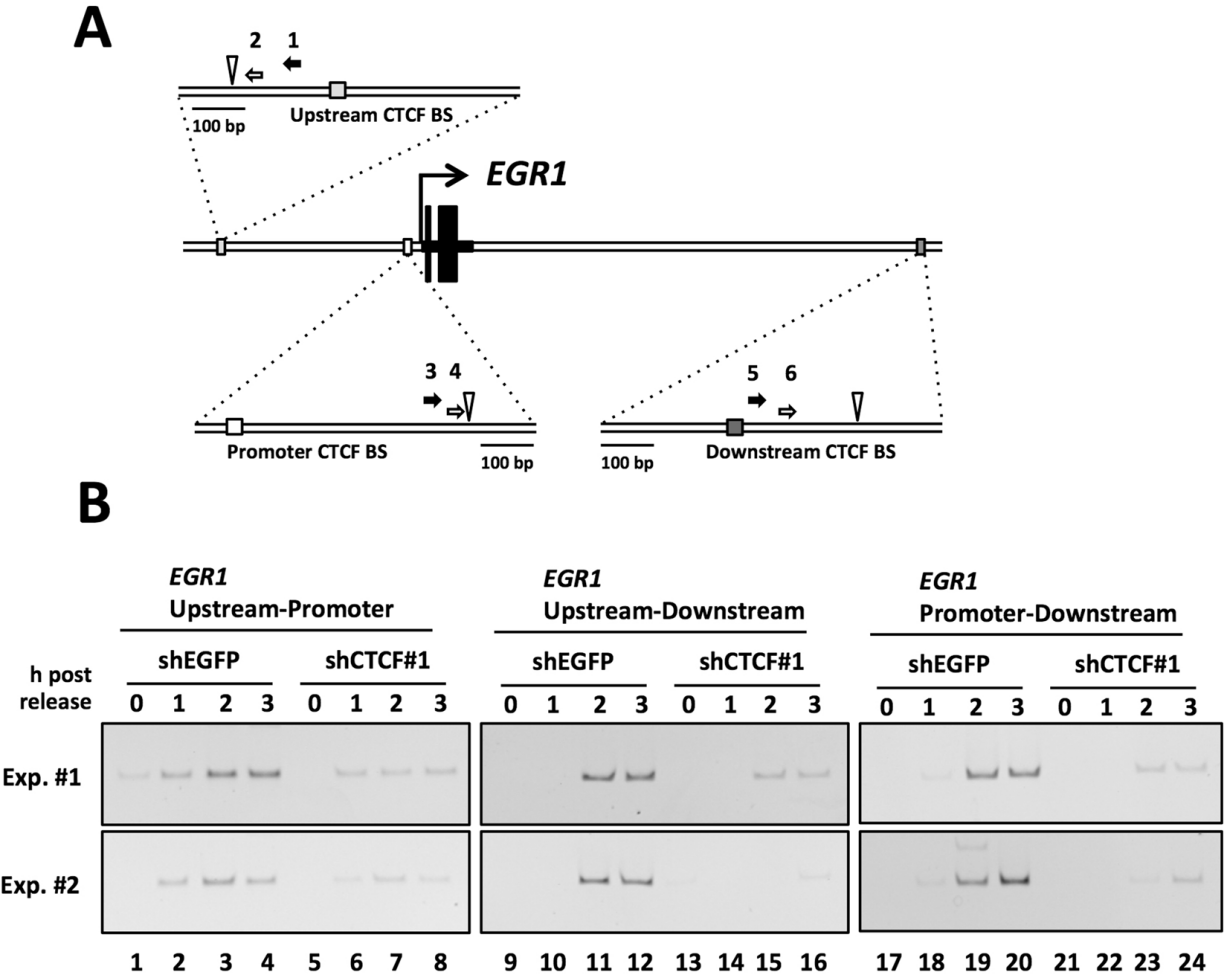
CTCF is involved in the organization of higher-order chromatin structure in the *EGR1* locus in early G1 phase. (**A**) Schematic diagram of the human *EGR1* gene locus and the PCR primers used for 3 C assays. Black and white arrows indicate primers for the first PCR and the second (nested) PCR, respectively to analyze interactions between upstream-promoter, upstream-downstream or promoter-downstream of the *EGR1* gene locus. The number of each primer is corresponding to that of in Table 1. The white inverted triangle indicates position of recognition sequence of restriction enzyme Dpn II. (**B**) HeLa S3 cells were transfected with shEGFP or shCTCF#1 expression vector. Cells were synchronized and arrested by nocodazole. Eary G1 cells were collected at indicated time points after nocodazole release, and then subjected to 3 C assay. The first round PCR was performed for 20 cycles, subsequently the second round PCR was performed by 30 cycles with nested primers. Results of two independent experiments (Exp. #1 and Exp. #2) are shown. The original gel images are presented in Supplementary Fig. S10.

It has been reported that CTCF plays a role in transcriptional regulation through interactions with transcription-related factors independently of higher-order chromatin structure formation^19,20^. To reveal the function(s) of CTCF in the *EGR1* transcription in early G1 phase, we used a luciferase reporter system to analyze the effect of CTCF bound to the promoter on *EGR1* transcription in early G1 phase. A DNA fragment corresponding to the promoter region between −720 bp and + 120 bp of the *EGR1* TSS was amplified by PCR and cloned into a luciferase reporter plasmid (p*EGR1* pro-720/+120 Luc). We also constructed a luciferase reporter plasmid containing a DNA fragment between −1358 bp and + 120 bp of the *EGR1* TSS (p*EGR1* pro-1358/+120 Luc) containing a promoter-proximal CTCF binding site located at the nucleotide position between −1227 bp and −1208 bp of the *EGR1* TSS. p*EGR1* pro-1358/+120ΔCTCF BS Luc has mutated nucleotide sequences of the promoter-proximal CTCF binding site (Figs 2A and S3A). The amounts of CTCF on p*EGR1* pro-1358/+120ΔCTCF BS Luc decreased to 25% of that on p*EGR1* pro-1358/+120 Luc (Supplementary Fig. S3B). However, no significant differences in the luciferase activity were found among cells transfected with plasmids p*EGR1* pro-720/+120 Luc, p*EGR1* pro-1358/+120 Luc, or p*EGR1* pro-1358/+120ΔCTCF BS Luc (Fig. 2B). These results indicate that, in the reporter plasmid-based assay system, the promoter-proximal CTCF binding site does not have any functions to the *EGR1* expression. This indicates that CTCF-mediated *EGR1* activation requires other CTCF binding sites in the *EGR1* locus in addition to the promoter-proximal binding site. Based on ChIP-sequence data from ENCODE project^18^, it is assumed that CTCF may bind to upstream and downstream of the *EGR1* gene locus (Supplementary Fig. S4). To confirm that CTCF binds to these loci, ChIP assays were performed using lysates prepared as described in Fig. 1. Figure 3B shows that CTCF binds to upstream (15 kb from the *EGR1* TSS) and downstream (38 kb from the TSS) sites in the *EGR1* gene locus in early G1 phase. Both the upstream and downstream CTCF binding sites showed similar increment patterns of CTCF binding as seen in the promoter-proximal CTCF binding site in early G1 phase (Fig. 1D). These results led us to examine the long-range chromatin interaction among the upstream, promoter, and downstream CTCF binding sites in the *EGR1* gene locus (Fig. 4).

### CTCF is involved in the formation of higher-order chromatin organization in the *EGR1* locus

To address whether long-range chromatin interactions exist in the *EGR1* locus, we performed 3 C analyses using primer sets indicated in Table 1 to identify a physical interaction between two separate genomic regions^21^. After crosslinking, the genomic DNA was digested with a restriction enzyme and subjected to ligation followed by PCR using primers corresponding to regions between 15 kb upstream and the promoter, 38 kb downstream and the promoter, and 15 kb upstream and 38 kb downstream (Fig. 4A). In control cells, amplified PCR products derived from any combinations among the *EGR1* upstream, promoter, and downstream regions were observed in a time-dependent manner after nocodazole release, indicating the close proximity of each these two separate regions forms in early G1 phase (Fig. 4B, lanes 1–4, 9–12, and 17–20). We did not observe any amplified PCR products using primers corresponding to the *Igf2* downstream and *EGR1* promoter regions (Supplementary Fig. S5), suggesting that the observed PCR signals in the *EGR1* locus reflect specific interactions between the two separate genomic regions. *Igf2* is a gene known to be regulated by CTCF and located on chromosome 11 which is different that of *EGR1*. Thus, *Igf2* and *EGR1* might be not close each other in the nucleus. The PCR products were reduced or not observed in CTCF KD cells (Fig. 4B, lanes 5–8, 13–16, 21–24), indicating that the long-range chromatin interactions observed in the *EGR1* locus were dependent on CTCF. From these results, it is possible that CTCF regulates *EGR1* transcription in early G1 phase as a chromatin organizer.

**Table 1:** Primer list for Figs 4 and 6.

		For first PCR		For second PCR	
For 3C-PCR	*EGR1* upstream	1	GCACCGTTTCAGGCAGGACACACACAAAAG	2	CTGACCCAGCTCAGAGAGGGAGAAAC
	*EGR1* promoter	3	CTGCTATACAGTGTCCCAAGAACCAAGTG	4	TCCCAGCGACACCCGGAAAGACACCGTGCC
	*EGR1* downstream	5	CACTTCCAGAGGACCAAGGTTGGGAGAGGA	6	TCCCAGTCCTTGCTAAGTGCCTTCATGTGC
	*Igf2* upstream	7	CTCACCTGTCTCCATAAAGCATGTGTCC	8	CCTTGCGTGGAAAATCTGTGGAATGTAG
	*Igf2* downstream	9	CTGCAAGGTCAGGGGACATTGTTCAGGG	10	GACATTGTTCAGGGTGGGAGGTGAGTGG
	*rRNA* forward	11	GCCCTTGCGGTGCTCCTGG		
	*rRNA* reverse	12	ACACACCACCGTTCGGCCTC		

**Figure 5:**
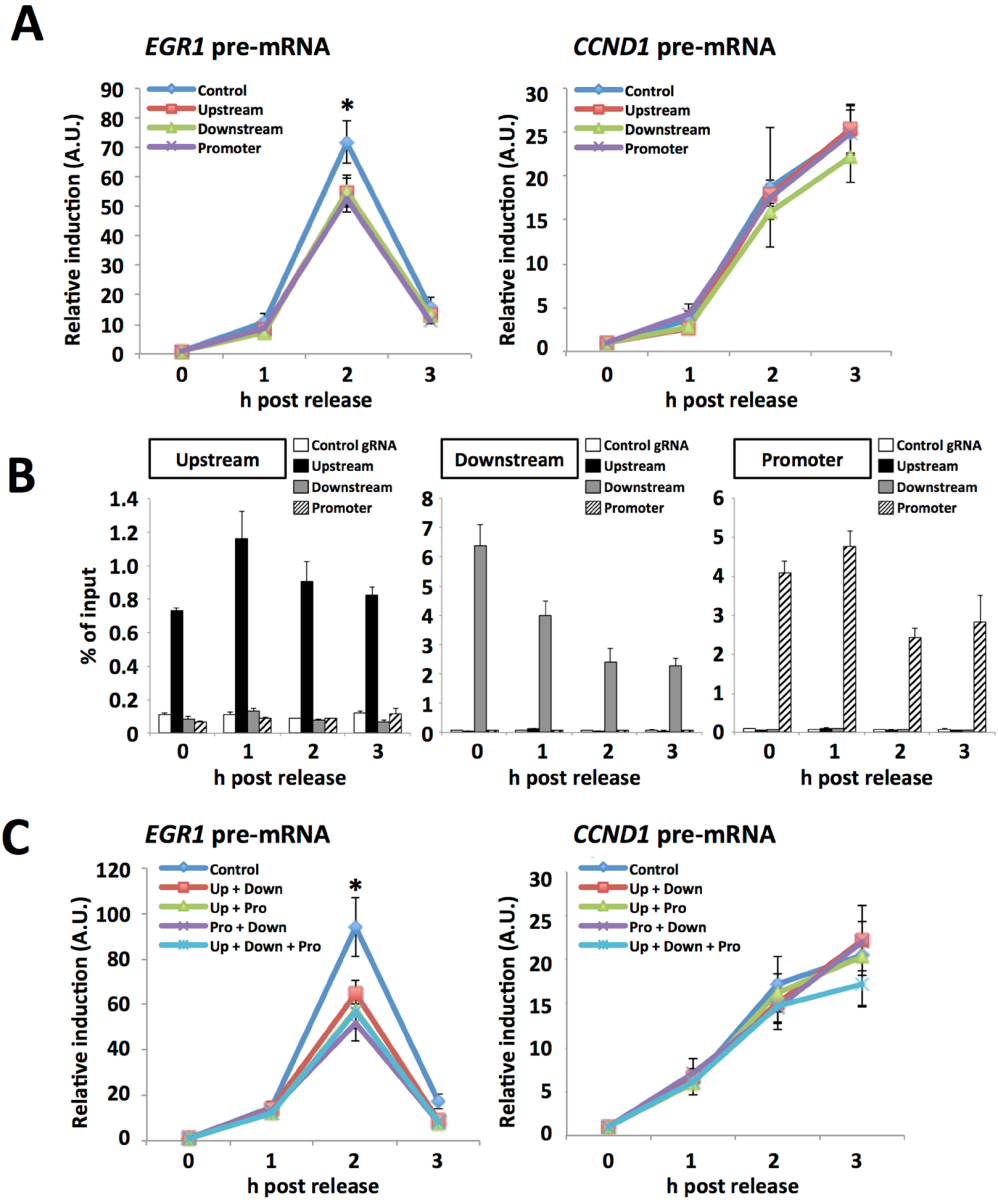
dCas9-mediated interference analysis. (**A**) dCas9-mediated specific interference of *EGR1* expression in early G1 phase. HeLa S3 cells were transfected with the plasmid expressing dCas9 with control gRNA (Control), gRNA against the 15 kb upstream (Upstream), gRNA against the 38 kb downstream (Downstream), and gRNA against the 1.2 kb upstream (Promoter). Cells were synchronized and arrested by nocodazole. Eary G1 cells were collected at indicated time points after nocodazole release. Total RNAs were extracted from collected cells and subjected to qRT-PCR using specific primer sets for *EGR1* pre-mRNA, *CCND1* pre-mRNA, and 28 S *rRNA*. The amounts of pre-mRNA were normalized by the amount of 28 S *rRNA*, and the relative expression levels were shown. Each error bar shows standard deviation of three independent experiments. Asterisks indicate a significant difference level (Student’s two tailed t-test, *P < 0.05). (**B**) ChIP analyses for a quantification of DNA associated with 3× FLAG tagged dCas9 and gRNA complexes. HeLa S3 cells were transfected with dCas9 and gRNA expression vectors and prepared early G1 cells as shown in (A), and ChIP assays were performed using anti-FLAG antibody. qPCR was performed with primer sets for amplification of 15 kb upstream (Upstream) and 38 kb downstream (Dpwnstream), and 1.2 kb upstream (Promoter) of the *EGR1* TSS. The relative amount of DNA co-immunoprecipitated with anti-FLAG antibody was shown as % of input. Error bar represents standard deviation (n = 2). (**C**) Simultaneous transfection of two or three kinds of gRNAs. HeLa S3 cells were transfected with the plasmid expressing dCas9 with control gRNA (Control), gRNAs against 15 kb upstream and 38 kb downstream (Up + Down), gRNAs against 15 kb upstream and 1.2 kb upstream (Up + Pro), gRNAs against 1.2 kb upstream and 38 kb downstream (Pro + Down), gRNAs against 15 kb upstream and 38 kb downstream and 1.2 kb upstream (Up + Down + Pro). Cells were collected and qRT-PCR analyses were performed as described in Fig. 5A. Each error bar shows standard deviation of three independent experiments. Asterisks indicate a significant difference level (Student’s two tailed t-test, *P < 0.05).

**Figure 6:**
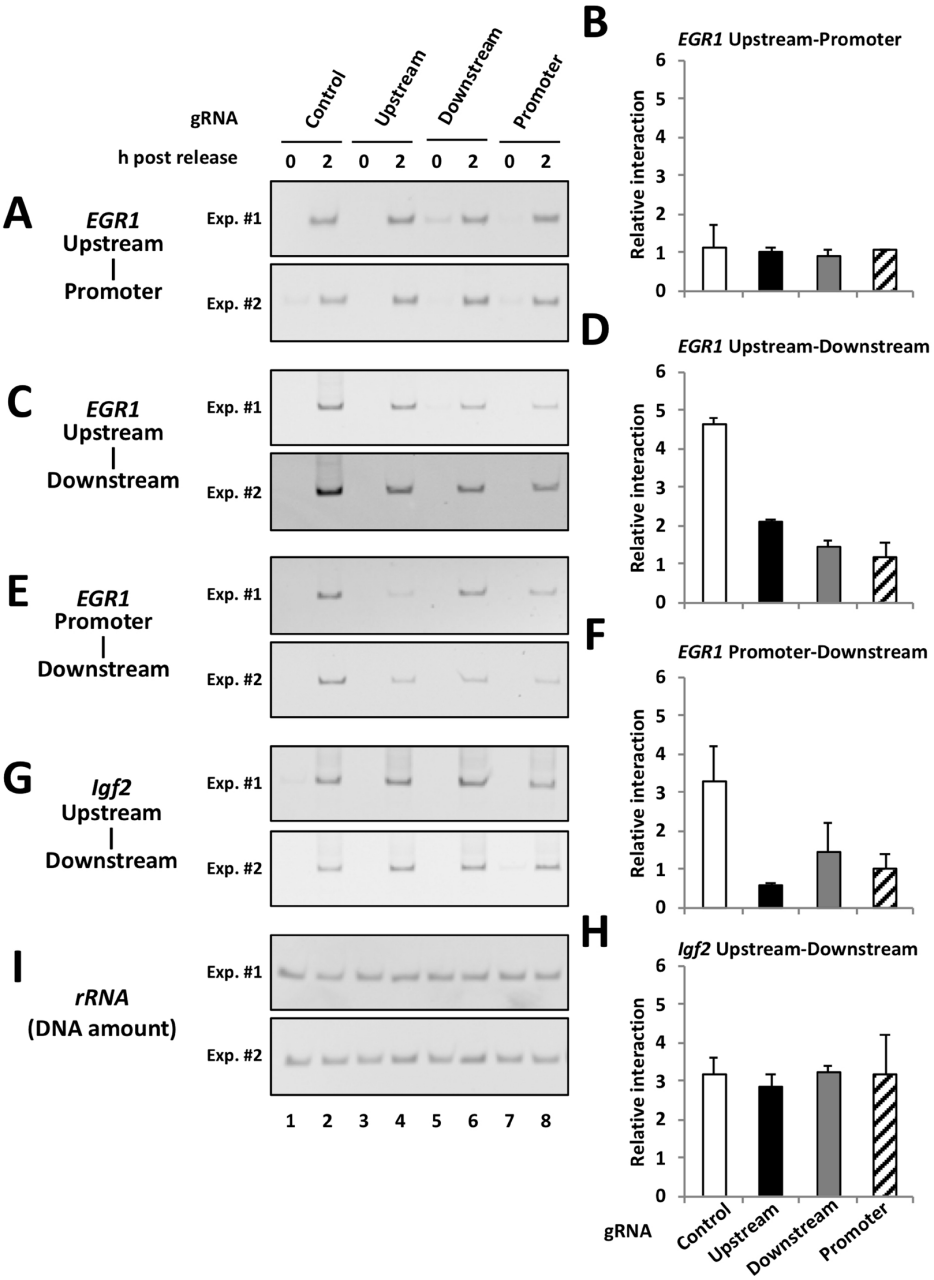
Effect of the dCas9-gRNA expression on chromatin interactions in the *EGR1* gene locus in early G1 phase. HeLa S3 cells were transfected with the plasmid expressing dCas9 with gRNA. Cells were synchronized and arrested by nocodazole. Eary G1 cells were collected at indicated time points after nocodazole release, and subjected to 3 C assay. The first round PCR was performed for 20 cycles, subsequently the second round PCR was performed by 30 cycles with nested primers. (**A**,**C**,**E**,**G**) PCR products were separated in a 6% polyacrylamide gel and visualized by staining with ethidium bromide. Results of 2 independent experiments (Exp. #1 and Exp. #2) are shown. (I) DNA amounts of each sample were normalized by amplification of *rRNA* gene locus which is not influenced from restriction enzyme digestion and ligation. (**B**,**D**,**F**,**H**) Representation of the relative interaction frequency under the dCas9 and gRNA expression conditions. The band intensity of each 3C-PCR product in 2 h post release samples represented in panel (**A**,**C**,**E**,**G**) was quantitatively measured with ImageJ and normalized using a standard curve derived from amplification of the standard template. Relative level of each 3 C interaction was graphed. Error bar represents standard deviation (n = 2). The interaction between upstream-downstream regions of *Igf2* gene locus was used as a control which is not influenced by gRNA-dCas9 against the *EGR1* gene locus. The original gel images are presented in Supplementary Fig. S11.

However, the results observed in the CTCF KD condition were potentially caused by an indirect effect due to the abundant existence of CTCF binding sites in whole genomic DNA. To investigate whether CTCF-mediated long-range chromatin interactions in the *EGR1* locus are required for transcription in early G1 phase, we developed a dCas9-mediated interference method to inhibit CTCF function in the *EGR1* locus. CRISPR-associated endonuclease Cas9 is recruited through RNA-DNA base pairing between Cas9-guide RNA (gRNA) and a specific target DNA sequence, resulting in the induction of a double-strand DNA break^22^. Because dCas9 has two alanine substitutions at amino acid positions 10 and 840 (aspartic acid and histidine, respectively), dCas9 has gRNA-dependent DNA binding activity but not nuclease activity. Thus, the dCas9-gRNA complex is recruited to a specific chromatin site without DNA damages^23^. To bring about interference of CTCF at each CTCF binding site, we designed gRNA sequences covering each of the CTCF binding sites. Cells were transfected with a plasmid construct expressing both 3× FLAG tagged dCas9 and gRNA. The gRNA targets were either a control nonspecific sequence (non-homologous with any CTCF binding sites in the *EGR1* locus), the upstream, downstream, or promoter proximal CTCF binding site in the *EGR1* locus (Supplementary Fig. S6). The cells were synchronized in prometaphase with nocodazole. At 0, 1, 2 and 3 h post release from nocodazole arrest, total RNAs were isolated and subjected to qRT-PCR for quantification of *EGR1* pre-mRNA. Compared with the cells expressing dCas9 with nonspecific gRNA, the cells expressing dCas9 with specific gRNA against the upstream, downstream, or promoter CTCF binding site showed reduced *EGR1* transcription in early G1 phase at 2 h post release (Fig. 5A). It is noteworthy that the effect of gRNA expression was specifically observed in transcription of *EGR1* but not *CCND1*, suggesting that the effect of gRNAs is highly specific for *EGR1* gene. The ChIP assays of dCas9 demonstrated that the gRNAs specifically interacted with each target site in the *EGR1* locus (Fig. 5B). To analyze whether dCas9 competes with CTCF for these binding sites, sequential ChIP analyses (termed re-ChIP) were performed. dCas9-bound chromatin was concentrated by immunoprecipitation with anti-FLAG antibody and then were subjected to further immunoprecipitation with control IgG, ani-CTCF, and anti-FLAG antibodies, respectively (Supplementary Fig. S7). We found that CTCF was not observed on the upstream (Supplementary Fig. S7, upper graph, lanes 5), the downstream (Supplementary Fig. S7, middle graph, lanes 8), and the promoter proximal CTCF binding sites (Supplementary Fig. S7, lower graph, lanes 11) by expressing gRNAs targeting to these sites, respectively. We also examined the *EGR1* transcription in cells transfected with two or three gRNAs targeting to the CTCF binding sites (Fig. 5C). The level of *EGR1* pre-mRNA but not *CCND1* pre-mRNA was reduced more than that with single gRNA transfected cells (Figs 5C and S8). To examine whether dCas9 expression also disrupts higher-order chromatin interactions at the *EGR1* locus, 3 C analyses were performed with cells expressing dCas9 and gRNAs against the CTCF binding sites in the *EGR1* locus (Fig. 6). We observed that the interactions of the upstream-downstream regions (Fig. 6C,D) and the promoter-downstream regions (Fig. 6E,F), but not the upstream-promoter regions (Fig. 6A,B), decreased by expressing dCas9-gRNA against CTCF binding sites in *EGR1* locus. Further, we also found that all three CTCF binding sites are required for the long-range interactions of the upstream-downstream and promoter-downstream regions. The CTCF binding sites located on upstream and promoter in the *EGR1* locus are on the forward strand (the coding strand of *EGR1*), while the downstream CTCF binding site of the *EGR1* locus is on the reverse strand (the template strand of *EGR1*). Because, it is reported that two CTCF binding sites located in convergent orientation interacts more often than that of located in the same orientation^24^. The 3 C interaction of the upstream-promoter region seems weaker compared with others (Fig. 6B). This might be due to the orientation of CTCF motif on each CTCF binding site in the *EGR1* locus. Notably, the interaction between the upstream and downstream regions of *Igf2* was not affected by dCas9-gRNAs for the *EGR1* locus (Fig. 6B, bottom graph). These results suggest that the dCas9-gRNA complexes interfere with the binding of CTCF to the *EGR1* upstream, downstream, or promoter CTCF binding site, and consequently the formation of 3 C interaction was disturbed. Therefore, it is concluded that CTCF mediates the long-range chromatin interactions among the CTCF binding sites in the *EGR1* locus. The interactions between upstream-downstream and promoter-downstream might be important for the expression of *EGR1* in early G1 phase.

## Discussion

EGR1 regulates several cellular processes such as cell proliferation and apoptosis as a transcription factor^2^. It has been reported that *EGR1* is transcribed in early G1 phase^9^. In general, transcription factors are the primary determinants of the transcription regulation. However, the molecular mechanism of the cell cycle-specific transcription of *EGR1* is not well understood. It has been reported that CTCF binds to the promoter-proximal CTCF binding site of *EGR1* and negatively regulates its transcription during myeloid cell differentiation or LPS-stimulated macrophages^11^. Here, we examined whether CTCF is involved in cell cycle-dependent transcription of the *EGR1*. We found that the expression of *EGR1* pre-mRNA in early G1 phase is reduced by knockdown of CTCF, suggesting an involvement of CTCF in the cell-cycle specific transcription of *EGR1* as a positive regulator (Fig. 1B). CTCF is known as a multi-functional protein^15^ and activates mRNA synthesis through its interaction with transcription-related factors such as the largest subunit of RNA polymerase II, RBP1/POLR2A^19^, or the general transcription factor TFII-I^20^. However, the promoter-proximal CTCF binding site did not activate the transcription from transiently transfected reporter plasmid in early G1 phase (Fig. 2). The inhibition of CTCF by shRNA or dCas9-gRNA resulted in reduced expression of *EGR1* pre-mRNA in early G1 phase (Figs 1 and 5) and impairment of chromatin-loop structures (Figs 4 and 6). The expression level of *EGR1* pre-mRNA showed sharp increment and decrement during early G1 phase (Figs 1B and 5A,C). During the period from 0 to 2 h post release from nocodazole block, CTCF-mediated higher-order chromatin structures within the *EGR1* gene locus might be required for its maximum transcription in early G1 phase. After that, the expression levels of *EGR1* pre-mRNA were decreased at 3 h post nocodazole release. However, binding levels of CTCF on each CTCF binding site (Figs 1C and 3B) and the formation of higher order chromatin structures in the *EGR1* locus (Fig. 4B) did not show the similar fluctuating patterns to that of the *EGR1* pre-mRNA levels. It might suggest that there is the inhibitory mechanism(s) of the *EGR1* expression which is controlled independently of CTCF-mediated higher-order chromatin structures. The gRNA against the promoter proximal CTCF binding site reduced the expression of *EGR1* in early G1 phase, suggesting that there is a functional significance of the promoter proximal CTCF binding site on the *EGR1* expression, even though it can not be reconstituted in transiently transfected plasmid lacking additional CTCF binding sites. These results suggest that higher order interactions between the promoter proximal CTCF binding site and other CTCF binding sites are important for its proper function. It has been reported that nucleosomes surrounding a CTCF binding site are well positioned^25^. Hence, we can not exclude the possibility that the local chromatin structure around the promoter proximal CTCF binding site might be important for its function in the *EGR1* locus because nucleosome positioning on transiently transfected DNA is usually different from that of native chromatin^26^. It has been reported that in mouse erythroblast cells, chromatin loop structure is generally disrupted in mitosis and increased sharply until early G1 phase (60–90 min post nocodazole release)^27^. Reformation of chromatin loop structures occur independently of its gene expression patterns during cell cycle, although the functional relationship between chromatin loop structures and its direct contribution to a gene expression is not clear^27^. Our findings suggest that the formation of higher order chromatin structures in the *EGR1* locus in early G1 phase may contribute to the expression of the *EGR1* pre-mRNA in early G1 phase. Enhancers regulate transcription of their target genes through the binding of transcription factors in a distance-independent manner^28^. It has been suggested that distal enhancers and their target promoters are closely positioned in three dimensional space through higher order chromatin structures to establish a microenvironment to regulate efficient gene expressions^29^. The enhancer database predicts a possibility of the presence of cell type specific putative enhancer elements in the human *EGR1* gene locus^30^. These putative enhancer elements may contribute to an optimal induction of *EGR1* in early G1 phase mediated by CTCF.

Not only *EGR1* but also other early G1 genes^9^ have CTCF binding sites in their gene loci (Supplementary Table S1), which suggests possible contribution of CTCF to expression of those genes via higher-order chromatin structure formation. However, it remains unclear whether our findings of CTCF involvement in the expression of the *EGR1* gene in the early G1 phase through higher-order chromatin structure formation could be expanded to other early G1 expression genes. Because of the technological limitation of PCR-based one-by-one analysis of higher-order chromatin interaction in being unable to handle multiple loci, several modified 3C-based methods have been developed to overcome this problem. Among these methods, the Hi-C technique^31^ is a practical methods to analyze genomewide chromatin interactions, especially in megabase-scale chromatin domain structures. Recently, it was reported that a modified Hi-C technique, called the *in situ* Hi-C protocol^24^, can be used to make a kilobase-scale high resolution interaction map. Analyses using such techniques may allow us to obtain more insights into the functional contribution of CTCF to the early G1 phase gene expression through higher-order chromatin structure formation.

## Methods

### Cell culture, transfection and synchronization

HeLa S3 cells were maintained at 37 °C in DMEM supplemented with 10% FBS. Cells were transfected with pU6-puro-shCTCF or pU6-puro-shEGFP^32^ plasmid using NEON transfection system (Thermo Fisher Scientific). After 20 h post transfection, puromycin was added in the medium at the concentration of 2 μg/ml, and cells were incubated further for 20 h. After selection in the presence of puromycin, mitotic cells synchronized with nocodazole were collected. Briefly, cells were treated with 2.5 mM thymidine (Sigma) in the culture medium for 16 h. Thymidine blocked cells were released into a fresh culture medium and incubated for 7 h, so that cells stayed in late S and G2 phases. Then, cells were treated with nocodazole (Sigma) at a final concentration of 165 nM and further incubated for 6 h. Mitotic cells were collected by gentle shaking of cell culture dishes. For release of cells from mitosis, nocodazole-arrested cells were reattached to poly-L-lysine coated culture dishes, then washed with PBS, and cultured for indicated periods.

### Preparation of plasmids

To generate pU6-puro-shCTCF#1 plasmid, which expresses 21 nucleotides-long hairpin-type shRNA against human *CTCF*, corresponding to the sequence between nucleotide positions 868 and 888 (NCBI Reference Sequence: NM_006565.3), 5′-CACCGTGTCTAAAGAGGGTCTTGTGGTGTGCTGTCCCGCAAGGCCCTCTTTAGACACTTTTTT-3′ and 5′-GCATAAAAAAGTGTCTAAAGAGGGCCTTGCGGGACAGCACACCACAAGACCCTCTTTAGACAC-3′ oligonucleotides were phosphorylated, annealed each other, and cloned into pU6-puro^32^ that had been digested with BspM I. Oligonucleotides corresponding to the sequence between nucleotide positions 2,819 and 2,839 (NM_006565.3), 5′-CACCGTAAATTATGGAGTGTTCTGATTGATATCCGTCAGAACATTCCATGATTTGCTTTTTT-3′ and 5′-GCATAAAAAAGCAAATCATGGAATGTTCTGACGGATATCAATCAGAACACTCCATAATTTAC-3′ were used to generate pU6-puro-shCTCF#2 plasmid. The human *EGR1* promoter was amplified by PCR using the genomic DNA extracted from HeLa S3 cells as template and cloned into PGV-B (TOYOBO). The primers 5′-TCCTCCCCCGCACTCCCGGTTC-3′ and 5′-GGGAACACTGAGAAGCGTGC-3′ (*EGR1*+120 reverse primer) were used for amplification of the *EGR1* promoter region harboring nucleotide positions from –720 to+120 relative to the TSS to make p*EGR1* pro-720/+120 Luc. The primers 5′-GCTCAGTTCGTGCTCACTGC-3′ and *EGR1*+120 reverse primer were used for amplification of the *EGR1* promoter region corresponding to nucleotide positions from –1358 to +120 to make p*EGR1* pro-1358/+120 Luc. A CTCF binding site (CTCF BS) corresponding to nucleotide positions from –1227 to –1208 was changed from GGAACCTCCAGGGGGCAGCA to GGAAGTTATAAGCTTCAGCA to make p*EGR1* pro-1358/+120ΔCTCF BS Luc. The sequencing information of nucleotide regions on p*EGR1* pro-1358/+120ΔCTCF BS Luc corresponding to the CTCF binding site are shown in Fig. S3A. To generate dCas9 cDNA, two amino acid point mutations were introduced into the Cas9 cDNA in pX330-U6-Chimeric_BB-CBh-hSpCas9 plasmid (a gift from Feng Zhang, Addgene plasmid #42230). Oligonucleotides containing a 20 nt guide sequence for the *EGR1* upstream CTCF binding site (5′-CACCGAGGCAGCTGCGCCACCTAGT-3′ and 5′-AAACACTAGGTGGCGCAGCTGCCTC-3′) and downstream CTCF binding site (5′-CACCGTTGTGCTGGTGACCACAAGG-3′ and 5′-AAACCCTTGTGGTCACCAGCACAAC-3′), and promoter proximal CTCF binding site (5′-CACCGAGTGGAGAGGGAACCTCCAG-3′ and 5′-AAACCTGGAGGTTCCCTCTCCACTC-3′) were phosphorylated, annealed, and ligated into Bbs I digested pX330-dCas9. To make a control template for 3 C assay, the DNA fragments corresponding to the *Igf2* gene locus were amplified by PCR and cloned into pBluescriptII SK + (pBS). The primers 5′-GGAGCTCACTCTAGTCTCCAAGGCTATT-3′ and 5′-CTCACCTGTCTCCATAAAGCATGTGTCC-3′ were used for amplification of the *Igf2* upstream region harboring nucleotide positions 11 kb upstream relative to the TSS to make pBS-*Igf2*-upstream. The primers 5′-CCTAGGATTGGAAGGACCCCATCATCTG-3′ and 5′-CTGCAAGGTCAGGGGACATTGTTCAGGG-3′ were used for amplification of the *Igf2* downstream region harboring nucleotide positions 102 kb downstream relative to the TSS to make pBS-*Igf2*-downstream. 5′-ends of PCR products were phosphorylated by T4 Polynucleotide Kinase and ligated into pBS digested with EcoR V.

### Antibodies

Antibodies used in this study were anti-CTCF (rabbit polyclonal)^33^, anti-α-tublin (DM1-A, Sigma), anti-EGR1 (B-6, Santa Cruz), anti-FLAG (M2, Sigma) antibodies and rabbit normal IgG (Millipore).

### Cellular extraction and western blot analysis

Toal cell lysats were prepared by lysing cells in sample loading buffer (62.5 mM Tris-HCl pH 6.8, 2% SDS, 10% glycerol, 100 mM β-mercaptoetanol, 0.01% bromophenol blue). Proteins were separated by 7.5% SDS-PAGE and subjected to western blot analysis. The signal was detected by Chemi-Lumi One L (Nacalai) and ImageQuant LAS4000 (GE Healthcare Life Sciences).

### Total RNA extraction and quantitative real-time PCR

Total RNA was prepared from cells using RNeasy mini kit (Qiagen) and RNase-free DNase I (Qiagen), and subjected to reverse transcription using ReverTra Ace reverse transcriptase (TOYOBO) with random 9 mer primers. Synthesized cDNA was used as template for quantitative real-time PCR (qRT-PCR) using FastStart SYBR Green Master (Roche) with specific primer sets, 5′-CAGCCCTACGAGCACCTGAC-3′ and 5′-ACTCCTGCGGTGAAGGACAG-3′ for *EGR1* pre-mRNA, 5′-CTGTGCATCTACACCGACAACTC-3′ and 5′-CTCGGAGGAGCAGATATGTCAGA-3′ for *CCND1* pre-mRNA, 5′-GCAATGAAGGTGAAGGCCGGCG-3′ and 5′-TAACACGTGCGCTCGTGCTCCACCTC-3′ for 28 S *rRNA*.

### ChIP assays

Cells were fixed by direct addition of formaldehyde to culture dishes at the final concentration of 0.5% and incubated at room temperature for 10 min. Cross-linking was terminated by the addition of glycine. Cells were washed with 125 mM glycine/PBS and collected by scraping. ChIP assays were carried out according to the protocol from Chromatin Immunoprecipitation Assay Kit (17–295; Millipore) with minor modifications. Elution buffer contains 1% SDS, 100 mM NaHCO_3_ and 10 mM DTT. The primers used here were as follows: 5′-AAACGGTGCCATATCCAGGCTG-3′ and 5′-TCCAGGGAGCGGAGGATGAG-3′ for *EGR1* gene 15 kb upstream region, 5′-GCTCAGTTCGTGCTCACTGC-3′ and 5′-GTTCCCTCTCCACTCCCAGC-3′ for *EGR1* gene 1.2 kb upstream region, 5′-CAGGCACCTCTTAATGCTTGTC-3′ and 5′-GCCAGGGAAAGTTTGTGCTG-3′ for *EGR1* gene 38 kb downstream region. To perform a specific PCR amplification from the promoter proximal CTCF binding site corresponding to the transfected reporter plasmid, the primers used here were as follows: 5′-CCAAACTCATCAATGTATCTTATGG-3′ and 5′-GGCTTGGCGGCTCGGTGCTG-3′. The former primer corresponds to the unique region on the plasmid. The latter primer corresponds to the promoter proximal CTCF binding site in *EGR1* gene. The quantitative PCR (qPCR) was performed using FastStart SYBR Green Master and Thermal Cycler Dice Real Time System (TaKaRa). The results were analyzed using Thermal Cycler Dice Real Time System Software Ver. 5.11. (TaKaRa).

### 3C assays

3C assays were performed essentially as described previously^34^. Cells were fixed by direct addition of formaldehyde to culture dishes at the final concentration of 1% and incubated at room temperature for 10 min. Cross-linking was terminated by the addition of glycine. Cells were washed with 125 mM glycine/PBS and collected by scraping. Cells were resuspended in Dpn II digestion buffer containing 0.3% SDS and incubated at 37 °C for 8 h. Triton X-100 was added at the concentration of 1.8%, and the samples were incubated at 37 °C for 1.5 h to sequester SDS. DNAs in samples were digested at 37 °C for 16 h with restriction enzyme Dpn II (New England Biolabs). The restriction enzyme was inactivated by the addition of SDS at the concentration of 1.6% and heating at 65 °C for 20 min. The samples were diluted by ligation buffer (66 mM Tris-HCl [pH 7.4], 5 mM MgCl_2_), to adjust concentration of DNA to 2.5 ng genomic DNA/μl each. Triton X-100 was added to 1% and incubated at 37 °C for 1 h to sequester SDS. T4 DNA ligase (TOYOBO), DTT, and ATP were added and incubated at 16 °C for 20 h followed by 30 min at room temperature. The samples were heated at 65 °C for 6 h to reverse cross-linking and incubated with Proteinase K (Nacalai) at 55 °C for 1 h. DNAs were purified from the samples by phenol/chloroform/isoamyl alcohol extraction and ethanol precipitation. A typical size distribution of genome DNA of uncut genome, Dpn II digested genome without ligation, and ligated genome DNA are shown in Fig. S5A. A control template for 3 C assay was prepared from the bacterial artificial chromosome (BAC) clone RP11–113M21 containing *EGR1* locus on human chromosome 5, pBS-*Igf2*-upstream, and pBS-*Igf2*-downstream. BAC DNA and plasmid DNA are mixed in equimolar amounts and digested with Dpn II. Digested DNA was randomly ligated by T4 DNA ligase. The random ligated DNA was purified and used as a standard template for semi-quantitative PCR. PCR was performed with primers shown in Table 1. Primer pairs for each PCR are shown in Table 2. The position of the restriction enzyme recognition site, CTCF motif, and position of primers in each of the nucleotide sequence is shown in Fig. S6. PCR products were separated in a 6% polyacrylamide gel and visualized by staining with ethidium bromide. Gel images were taken by Gel Documentation System (ATTO). Band intensities were measured by ImageJ^35^.

**Table 2:** Primer pairs for 3C-PCR.

		First PCR	Second PCR
3 C interaction	*EGR1* upstream-promoter	1 + 3	2 + 4
	*EGR1* upstream-downstream	1 + 5	2 + 6
	*EGR1* promoter-downstream	3 + 5	4 + 6
	*EGR1* promoter-*Igf2* downstream	3 + 9	4 + 10
	*Igf2* upstream-promoter	7 + 9	8 + 10

### Luciferase assays

HeLa S3 cells were transfected with p*EGR1* pro-720/+120 Luc, p*EGR1* pro-1358/+120 Luc, p*EGR1* pro-1358/+120ΔCTCF BS Luc plasmids together with pRL-SV40 (Promega) plasmid. Renilla luciferase expression plasmid pRL-SV40 was used as a normalizer. Transient transfection of plasmid DNA for reporter gene assay was performed using GeneJuice (Novagen). Cells were collected at 0, 1, 2, and 3 h post release from nocodazole treatment as described previously. The cells were lysed with lysis buffer (Renilla luciferase lysis buffer, Promega). The cell lysates were mixed with a firefly luciferase substrate (Promega) or rennilla luciferase substrate (Promega), and each luciferase activity was measured by Lumat LB9506 (BERTHOLD). Each value was normalized by the renilla luciferase activity.

### Time-lapse analysis

HeLa S3 cells were transfected with shEGFP or shCTCF#1 expression plasmid and cell cycle were synchronized as described above. Nocodazole arrested cells were collected and reattached to a poly-L-lysine coated 12-well plate. The plate was put in a chamber of ARRAYSCAN XTI (Thermo Fisher Scientific) and maintainted at 37 °C in 5% CO2. Then, cells were washed with PBS, and time-lapse images were collected by ARRAYSCAN XTI.

## Electronic supplementary material

Supplementary Figures
