# Supplementary material for: Involvement of CTCF in transcription regulation of EGR1 at early G1 phase as an architecture factor
Source: Sci Rep. 2019 Jan 23;9:329. doi: 10.1038/s41598-018-36753-x (PMC6344568; doi:10.1038/s41598-018-36753-x)
Supplement: Supplementary file 1 — Supplementary Figures [file 41598_2018_36753_MOESM1_ESM.pdf]

Involvement of CTCF in transcription regulation of EGR1 at early G1 phase as an architecture factor

Takeshi Sekiya[1], Kohsuke Kato[1,2], Atsushi Kawaguchi[1,2], Kyosuke Nagata[2,*]

Department of Infection Biology, [1]Graduate School of Comprehensive Human Science, [2]Faculty of Medicine, University of Tsukuba, Tsukuba, Japan

Table of contents

Supplementary Figure S1
Supplementary Figure S2
Supplementary Figure S3
Supplementary Figure S4
Supplementary Figure S5
Supplementary Figure S6
Supplementary Figure S7
Supplementary Figure S8
Supplementary Figure S9
Supplementary Figure S10
Supplementary Figure S11
Supplementary Table S1

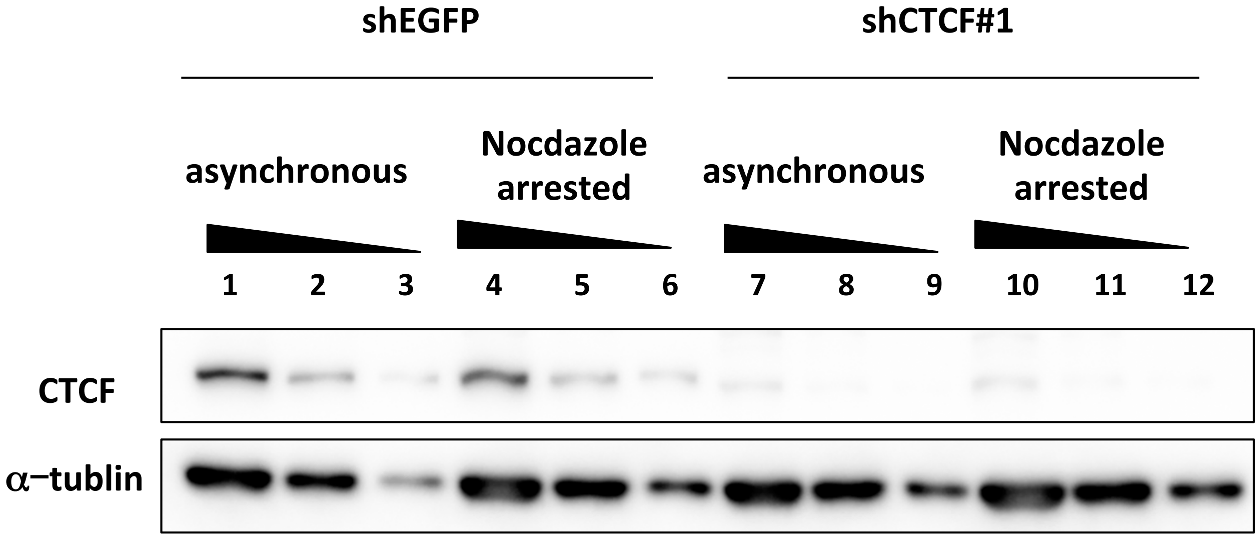

Figure S1. Cell cycle synchronization did not change the expression of CTCF. HeLa S3 cells were transfected with shEGFP expression plasmid as a control or shCTCF#1 expression plasmid. Asynchronously cultured cells or nocodazole arrested cell were collected. Lysates from desired cell numbers (1×10^5, 0.5×10^5, and 0.25×10^5) were subjected to western blot analyses using anti-CTCF and anti-α-tublin antibodies.

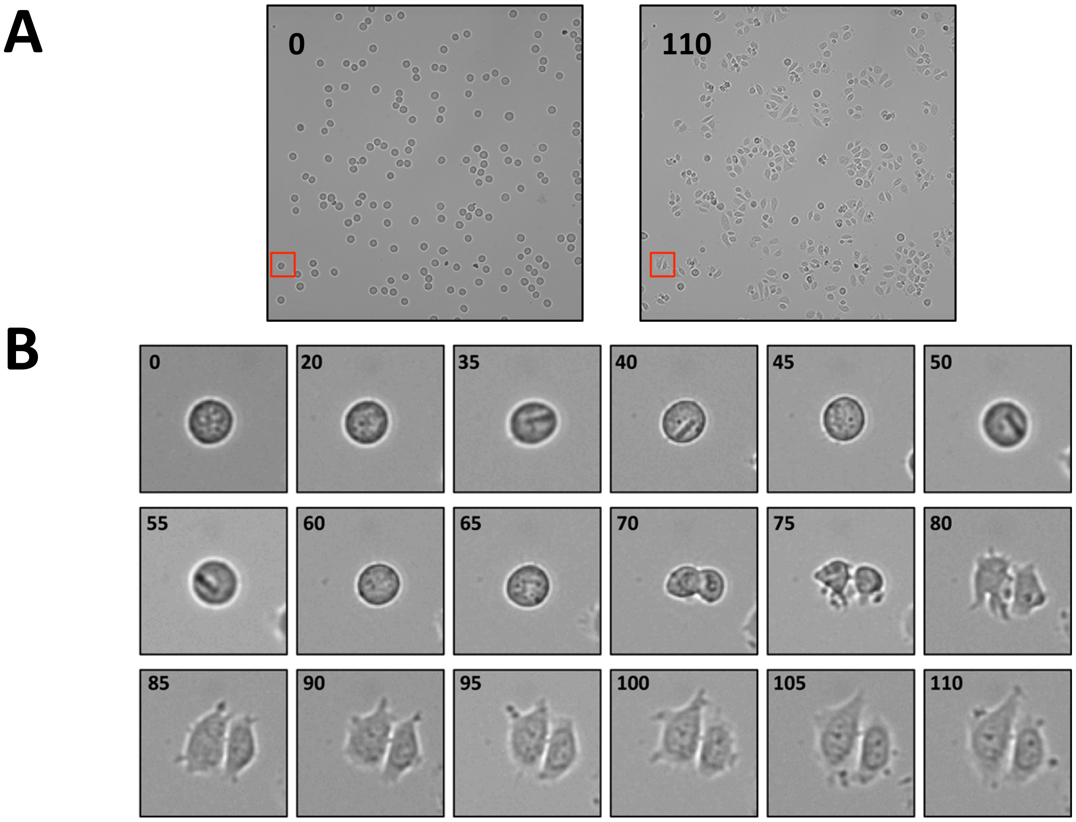

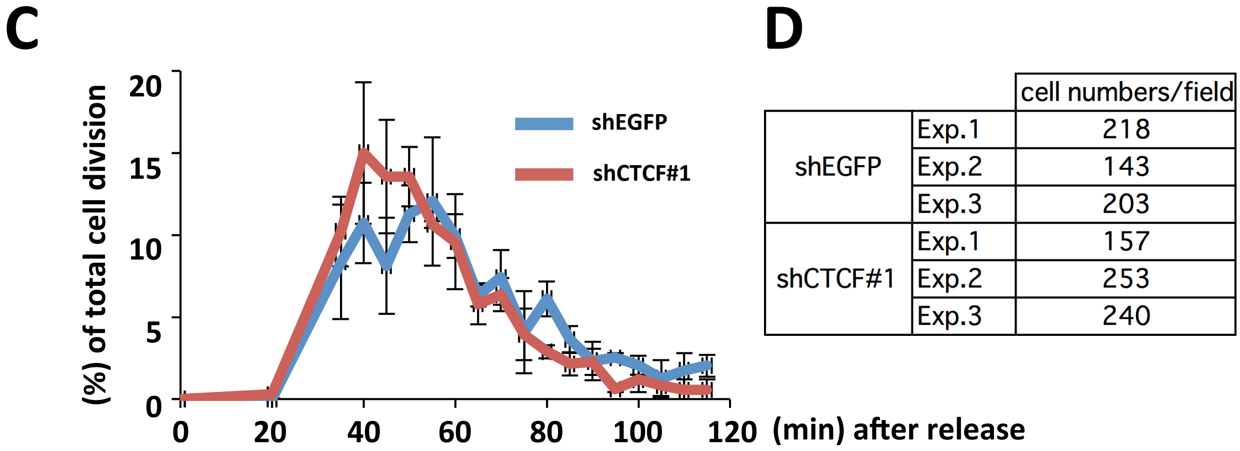

D

		cell numbers/field
shEGFP	Exp.1	218
	Exp.2	143
	Exp.3	203
shCTCF#1	Exp.1	157
	Exp.2	253
	Exp.3	240

Figure S2. Cell cycle progression for exit of mitosis in control and CTCF KD cells. HeLa S3 cells were transfected with shEGFP or shCTCF#1 expression plasmid and mitotic cells were collected as described in methods. For release of cells from mitosis, nocodazole-arrested cells were reattached to a poly-L-lysine coated culture plate, then washed with PBS, and cultured for indicated periods. The plates were subjected to time lapse imaging analyses of cell cycle progression. Cell images were collected at 0, 20, 35, 40, 45, 50, 55, 60, 65, 70, 75, 80, 85, 90, 95, 100, 105, and 110 minutes after nocodazole release. (A) An example of cell image. Every fields contained more than 100 cells/per field. A left panel showed a image at 0 minute post nocodazole release and a right panel showed a image at 110 minutes post release of the same field. (B) Magnified images of a single cell framed by a red square on (A). In this example, the image of 70 minutes post release was quantified as a cell division event. (C) Each cell division event was calculated and the ratio of event occurred at each time point was plotted. The data represent mean values with standard deviations from three independent biological experiments. (D) Cell numbers counted in each experiment were indicated.

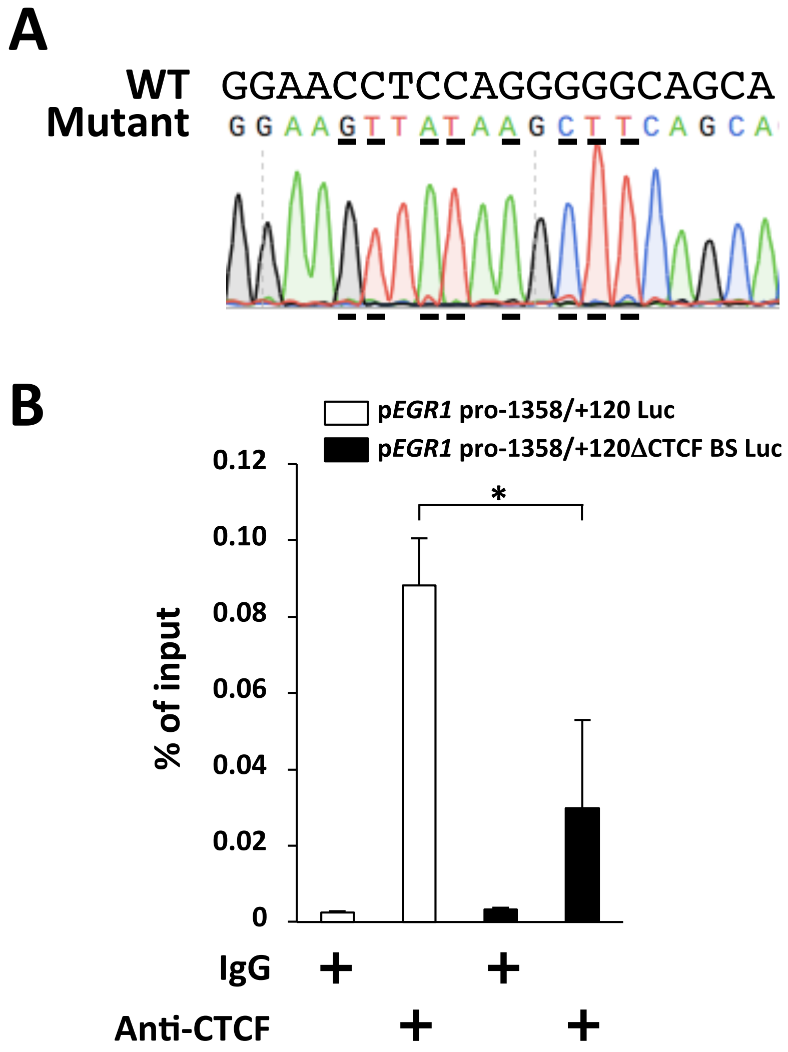

Figure S3. Related to Fig.2

(A) The sequence information of the p*EGR1* pro-1358/+120ΔCTCFBS Luc reporter plasmid. "WT" shows original sequence of the promoter-proximal CTCF binding site corresponding to -1227 bp to -1208 bp form the *EGR1* TSS. "Mutant" shows the nucleotide sequence and peak patterns of sequencing analysis corresponding to the promoter-proximal CTCF binding site on the plasmid. Black bars show mutated nucleotides. (B) The amount of CTCF on transiently transfected plasmids. HeLa S3 cells were transfected with p*EGR1* pro-1358/+120 Luc or with p*EGR1* pro-1358/+120ΔCTCFBS Luc reporter plasmid. Asynchronously cultured cells were fixed with formaldehyde and ChIP assays were performed using anti-CTCF antibody and rabbit normal IgG. The relative amount of DNA co-immunoprecipitated with each antibody was shown as % of input. Data information: Each histogram shows standard deviation of three independent experiments. Asterisks indicate a significant difference level (Student's two tailed t-test, *$P < 0.02$).

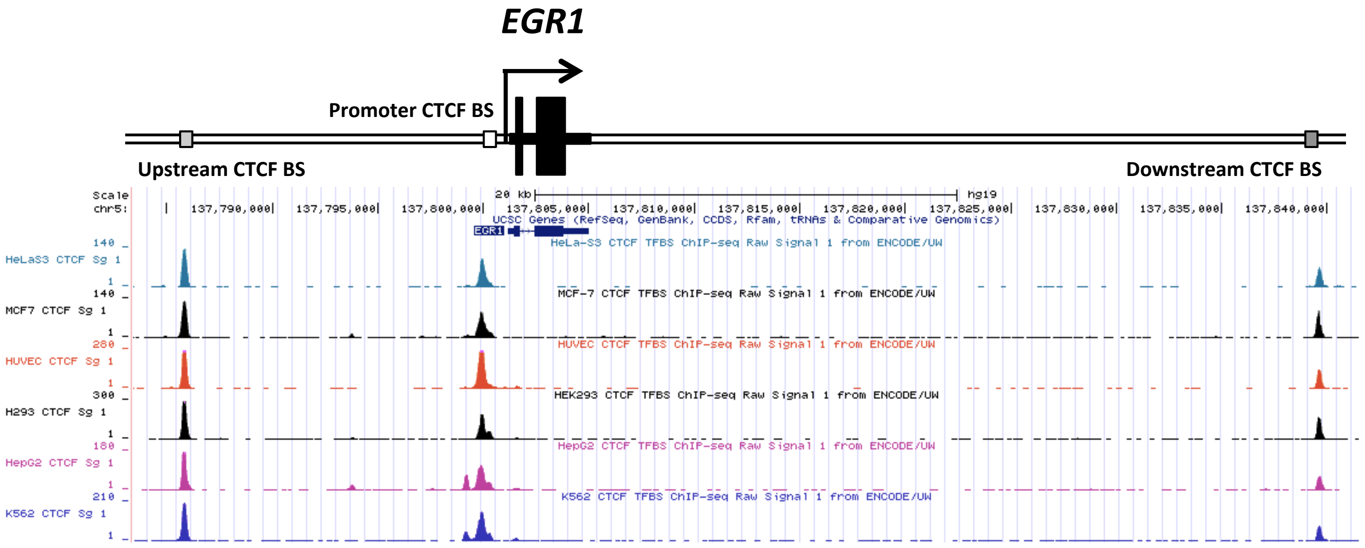

Figure S4. Visualized CTCF enrichment patterns of 6 human cell lines in chromosome 5; 137,783,400-137,841,500. CTCF ChIP-sequence data sets of HeLa S3, MCF7, HUVEC, HEK293, HepG 2, and K562 cells (Part of CTCF Binding Sites by ChIP-seq from ENCODE/University of Washington) are shown on the UCSC genome browser. Schematic diagram of *EGR1* and CTCF binding sites are shown.

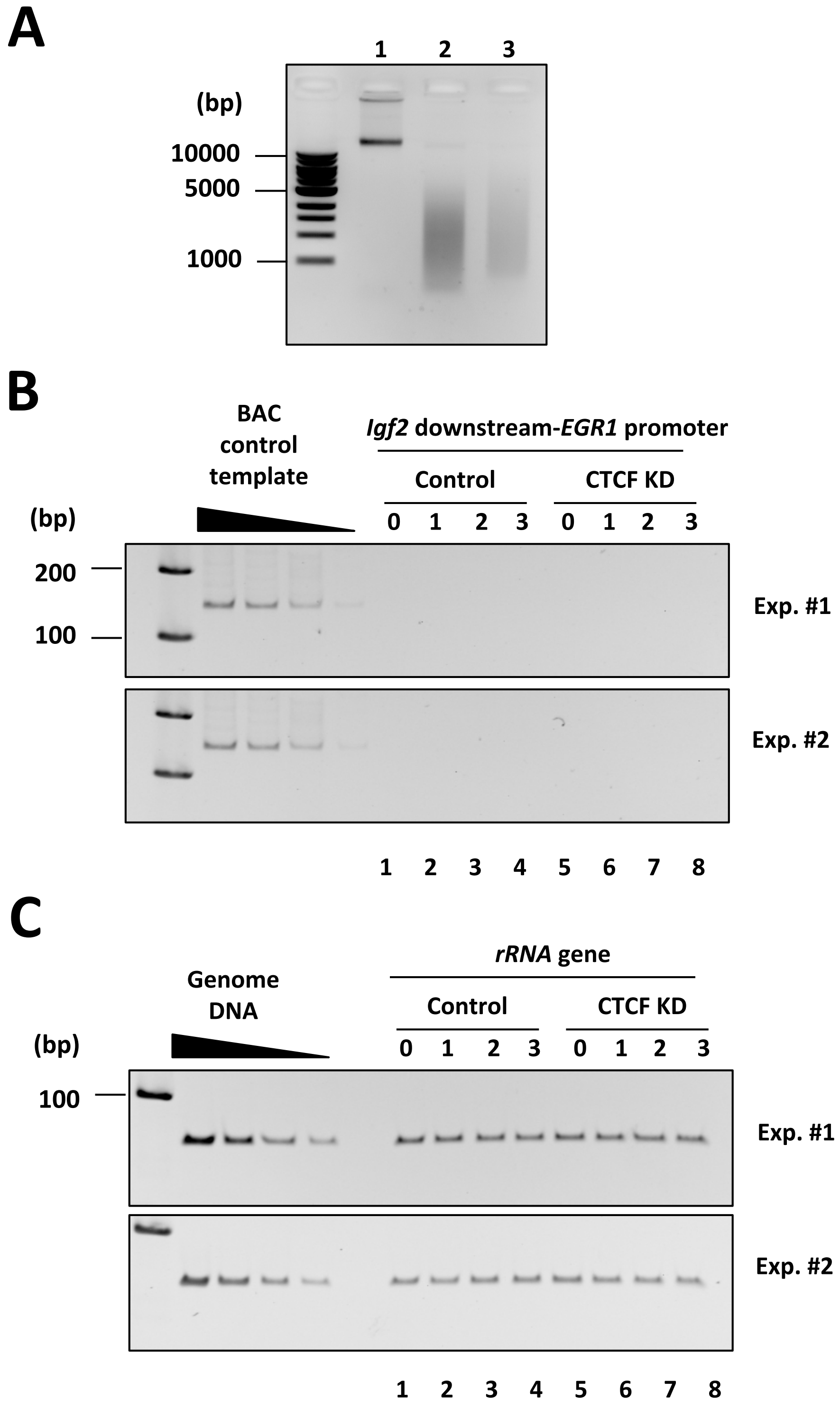

Figure S5. Related to Fig. 4.
(A) A typical agarosegel electrophoresis image showing undigested (lane1), digested (lane2) and ligated DNA (lane3) used for 3C assay. (B) *Igf2* downstream and *EGR1* promoter primer combination was used as a control for evaluation of random ligation. Results of two independent experiments are shown. (C) PCR products of *rRNA* gene locus, which is not influenced from restriction enzyme digestion and ligation, are shown. Results of two independent experiments are shown.

A Upstream CTCF binding site

hg19 chr5: 137785611-137785930

ACTATCTTCAATTTCCGCCAGTGCACGATCACCCCCCTCCCACATCCCCCCAGTTTCTCCCTCT
CTGAGCTGGGTCAGGTCCGTCGGGAGGAGGAGGGCATCTCCTGTCCTTACCGGACAGCGGCTTT
TGTGTGTGTCCTGCCTGAAACGGTGCCATATCCAGGCTGGAGGCGGCGAGGCGTGGGAGGGATG
CCTGCGAGGATGGAAAAGCCCACTAGGTGGCGCAGCTGCCTCCCTATGTGGGATTCCCCGGCCC
CGCGGACAATCCTCTCCCGCGGCCAGTAGGCTCCAGGTTTGGTGAGCAGAGTGCCTCATCCTCC

B Promoter CTCF binding site

hg19 chr5: 137785611-137785930

CCAGCGCCGCATCCGGGAGGAGGAGCGAGGAGGCGGCGGAAGAGCCCGCGCGGCCGGAGTCCGG
GGCTGGGAGTGGAGAGGGAACCTCCAGGGGGCAGCACCGAGCCGCCAAGCCGGTCCTCTCTTCG
CGCCCAGCCCGGGGTCCCCAGACAGCCCATAGGGAAGCCCCTCTTTCGGATTCCCGCAGTGTGG
GCCGGCCCTCCACCTGGACTGGATAAAGGGGGGAAAGTGACCCCTCACCACAAGGACCATTATC
TCCTGGTGAGAACAAGAATCAGGCCTCTCTTGGGGCAATCAGCTTCCCCACTTCGGTCCCCCAA
AGGTGGGCTCTTTGCCGGCGGGGACTAGGGAACAGCCTTTCGGTTCCGGGGGAGCACAGGGGAC
CCCAGGCACCAGCAGCCCCATCCCACCGACAGGTGGCAGAGGCAAGGCAGCTCACTGCTATACA
GTGTCCCAAGAACCAAGTGGCCGTGACTTCCTATCCTCAATTTCCCAGCGACACCCGGAAAGAC
ACCGTGCCATAGATCGAGGCCCGGGGTCAAGGCCCCGCCTCTCCTGGGCGGCCCCTGCCCAGGC

C Downstream CTCF binding site

hg19 chr5: 137839535-137839727

CAGGCACCTCTTAATGCTTGTCAGAAATCCCCCCTACTTCAGATGAAGCAAATTTCTTCCAGTC
AGCATCCTTAACTCTATACCACCACCTTGTGGTCACCAGCACAAACTTTCCCTGGCCACTTCCA
GAGGACCAAGGTTGGGAGAGGAAGAACCATGAGTAGCTGCCATCCCAGTCCTTGCTAAGTGCCT
TCATGTGCCTTACTCCATTTAATCCTTATTACAGGTGGTGAAACTGCTACTCAAAGACATGGAA
TAATTTGCCAGCCTCTCAGGTGGCGCCAGGATTTCTACCTCAGACCTCAGAGATCCTGCTTCCT

Figure S6. Related to Figs.4 and 6.
The nucleotide sequence of CTCF binding motifs located on 15 kbp upstream (A), 1.2 kbp upstream (B), and 38 kbp downstream (C) from the *EGR1* TSS are shown respectively. The recognition sequence of the restriction enzyme Dpn II in each CTCF binding site is highlighted by green. Position of primers for 3C assay are indicated by red arrows (for 1st PCR) and blue arrows (for nested PCR) . The CTCF motifs highlighted yellow are the forward orientation and the motif highlighted blue is the reverse orientation. The Each target sequence of gRNA is underlined and complement sequence of protospacer adjacent motif (PAM) is indicated by red characters.

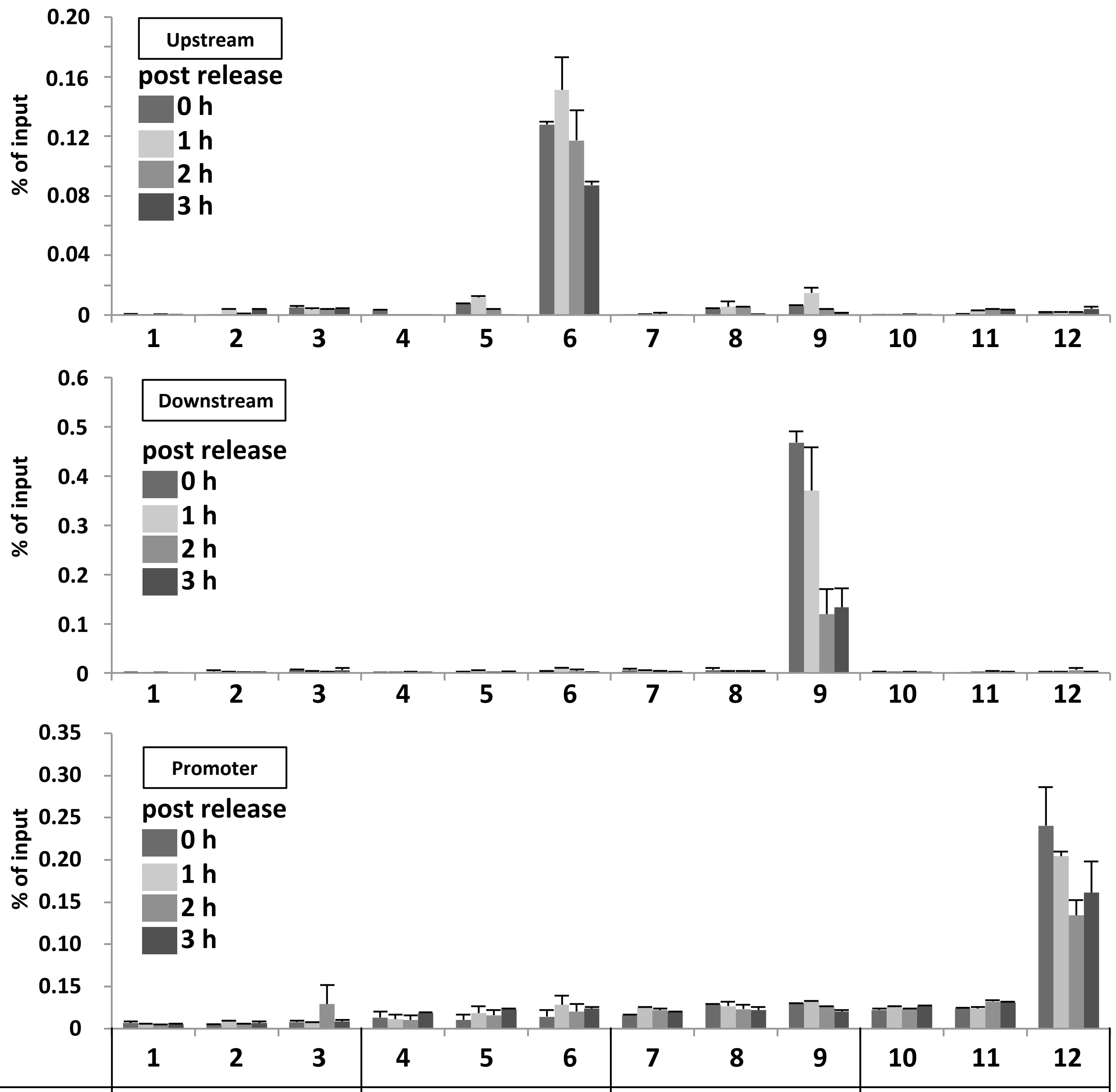

2nd ChIP	IgG	CTCF	FLAG	IgG	CTCF	FLAG	IgG	CTCF	FLAG	IgG	CTCF	FLAG
1stChIP	FLAG			FLAG			FLAG			FLAG		
gRNA	control			Upstream			Downstream			Promoter		

Figure S7. ReChIP assay. Cell preparations and ChIP assays were performed as described in Fig. 5B. The immunoprecipitated complexes with anti-FLAG antibody were then subjected to the second ChIP with rabbit normal IgG (lanes 1, 4, 7, and 10), anti-CTCF (lanes 2, 5, 8, and 11) or anti-FLAG (lanes 3, 6, 9, and 12) antibodies. qPCR was performed with primer sets for amplification of 15 kbp upstream (Upstream, upper graph) and 38 kbp downstream (Downstream, middle graph), and 1.2 kbp upstream promoter-proximal (Promoter, lower graph) regions of the *EGR1* gene TSS. Error bar represents standard deviation (n = 2).

Figure S8

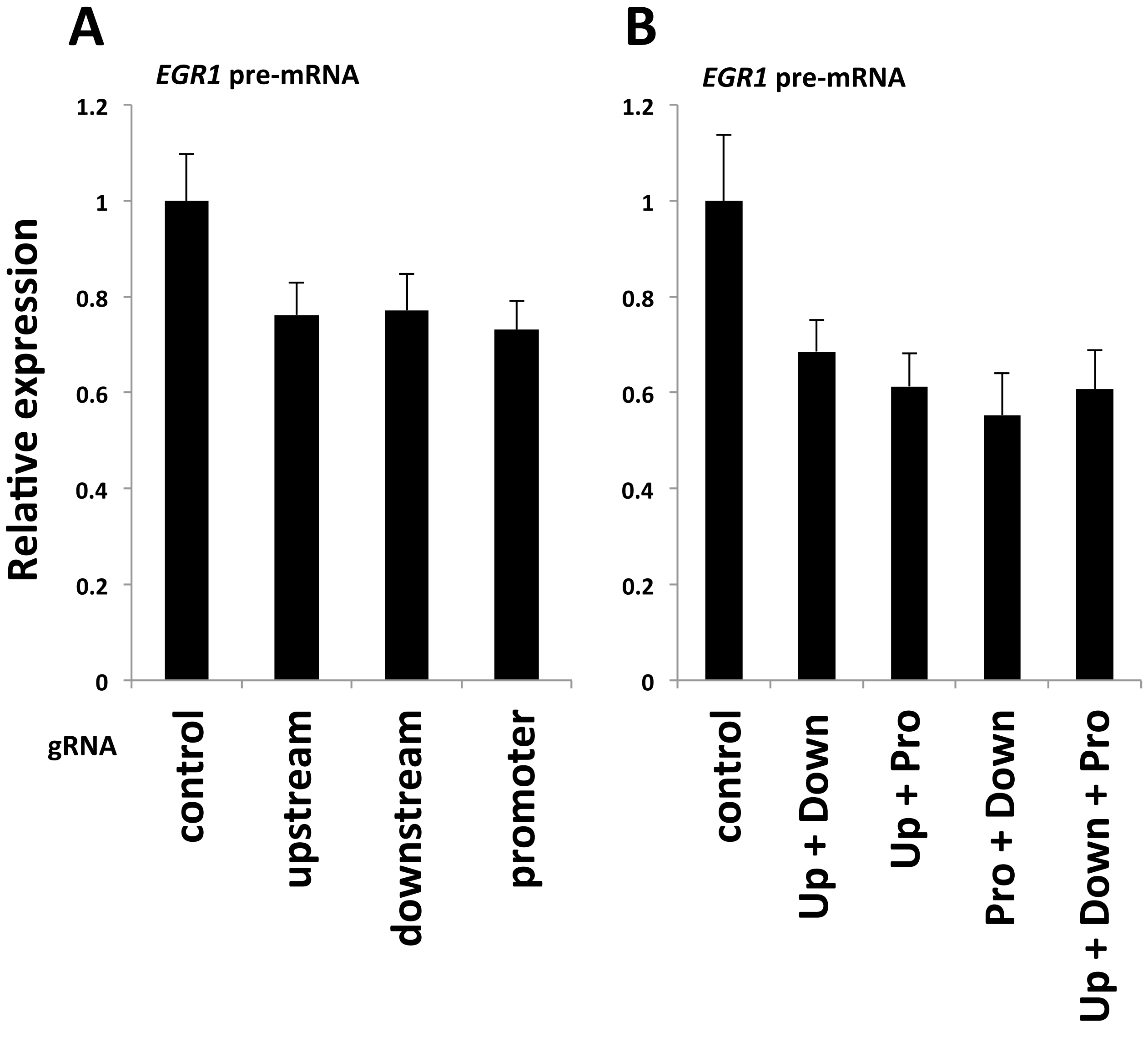

Figure S8. Related to Fig. 5. The expression levels of *EGR1* pre-mRNA at 2 h post release were indicated as relative expression to the control gRNA transfected cells. (A) Relative expression levels of *EGR1* pre-mRNA at 2 h post release from Fig. 5A. (B) Relative expression levels of *EGR1* pre-mRNA at 2 h post release from Fig. 5C.

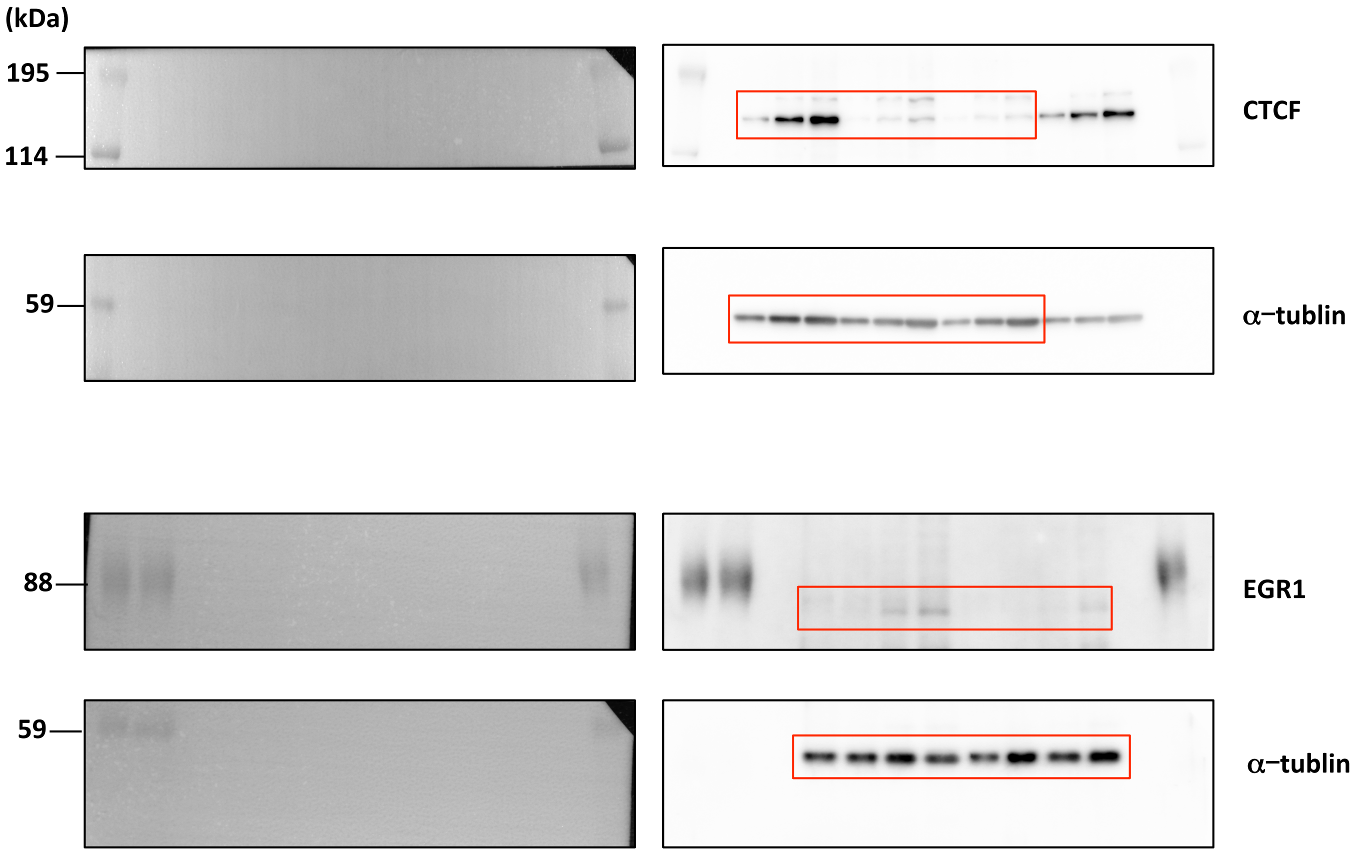

Figure S9. Uncropped images of blots presented in the main Figs 1A and 1C. Left panels show membrane images and right panels show chemiluminescence images. Red lines delineate regions used in Figs 1A and 1C.

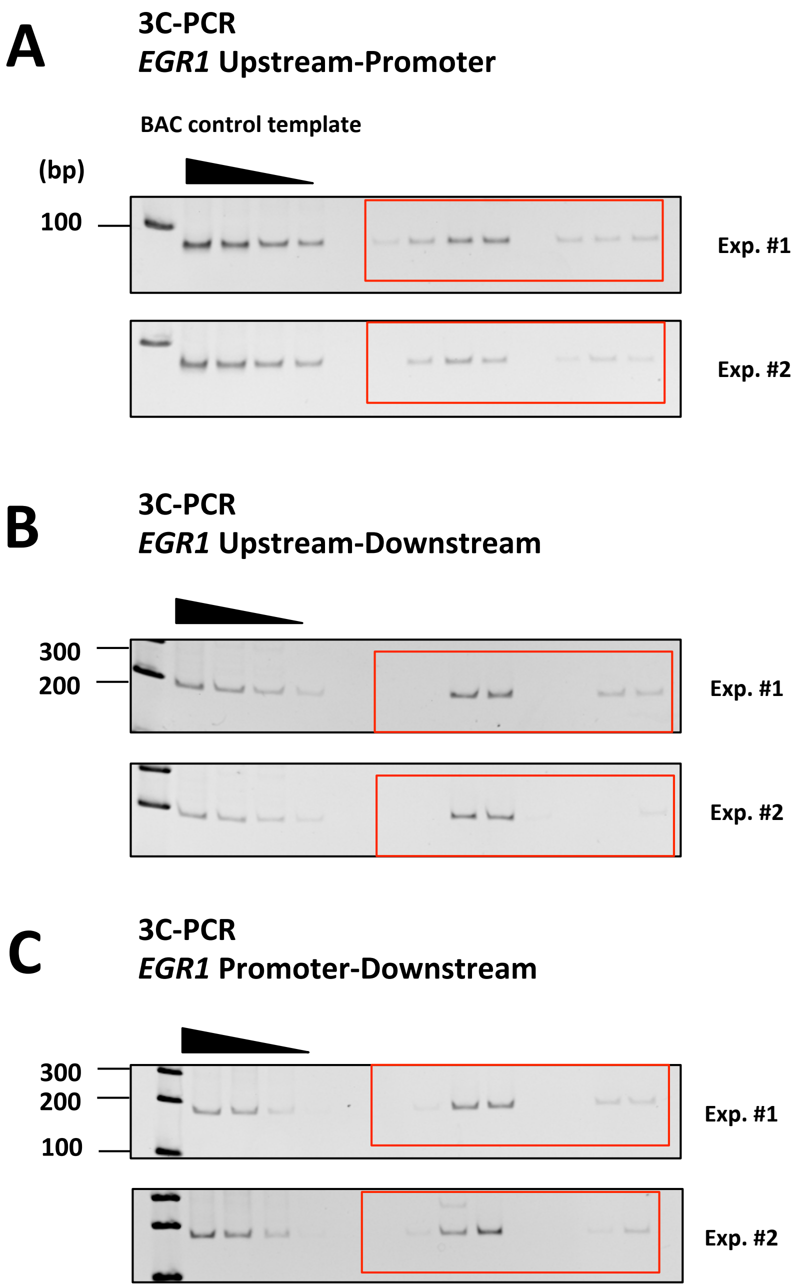

Figure S10. Uncropped images of 3C-PCR bands separated in polyacrylamide gel presented in the main Fig. 4B. (A) *EGR1* Upstream-Promoter. (B) *EGR1* Upstream-Downstream. (C) *EGR1* Promoter-Downstream. Red lines delineate regions used in Fig. 4B.

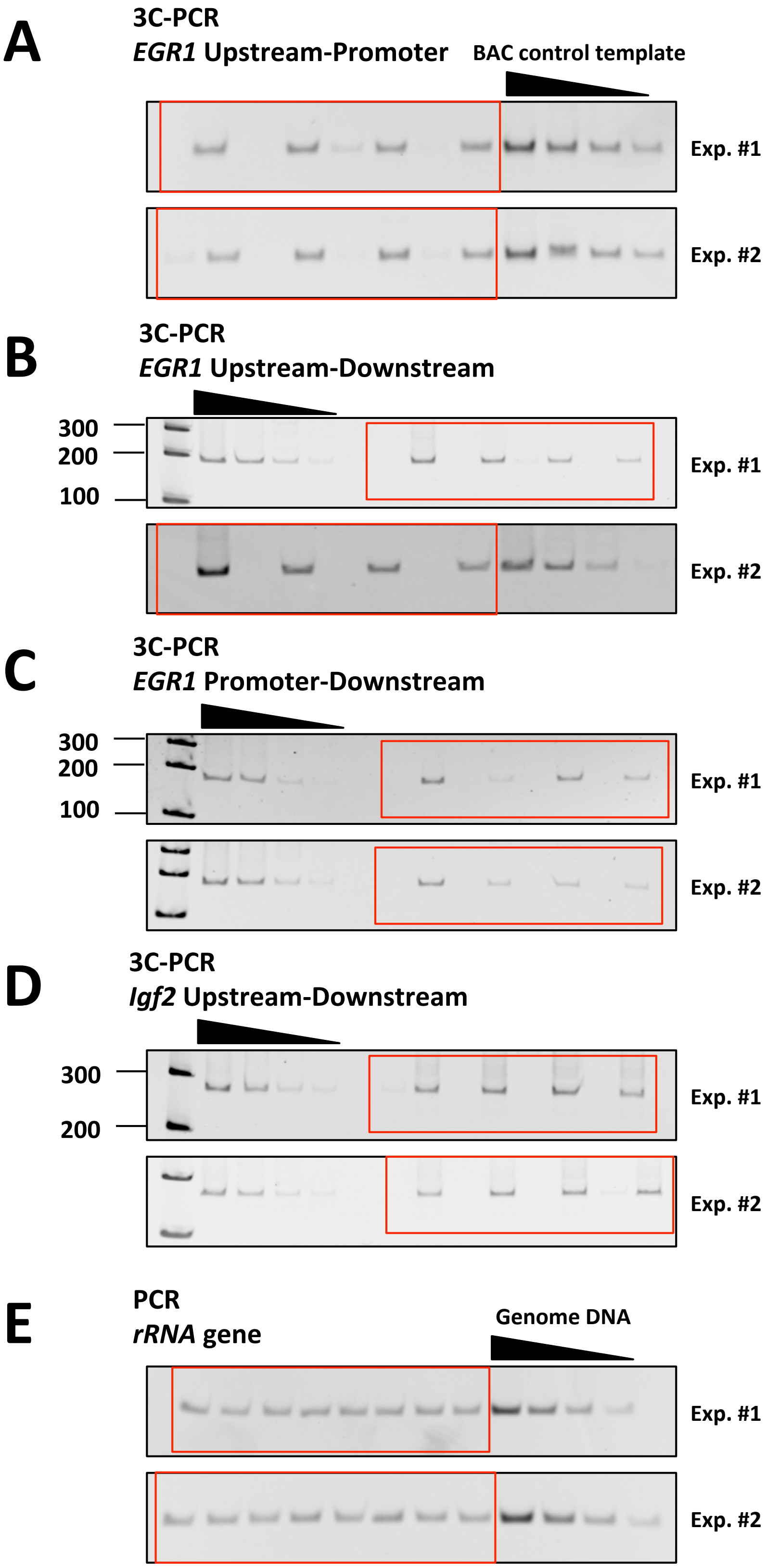

Figure S11. Uncropped images of 3C-PCR bands separated in polyacrylamide gel presented in the main Fig. 6. (A) *EGR1* Upstream-Promoter. (B) *EGR1* Upstream-Downstream. (C) *EGR1* Promoter-Downstream. (D) *Igf2* Upstream-Promoter. (E) PCR products of *rRNA* gene locus. Red lines delineate regions used in Fig. 6.

Supplemental Table S1

List of 77 genes, which are expressed in early G1 phase
and CTCF binding was observed in ENCODE ChIP-seq date.

1	AHI1	41	IDH1
2	AKAP13	42	LIMCH1
3	ANK3	43	MAN1A2
4	ANKH	44	MAN1C1
5	ANKRD11	45	MAT1A
6	ANKRD12	46	MMP28
7	AP4E1	47	MYADM
8	ARHGEF26	48	NMNAT3
9	ASH1L	49	OGT
10	ASXL1	50	PAFAH1B2
11	ATF3	51	PAX2
12	BBX	52	PGM5
13	BCR	53	PIGN
14	BRD2	54	PLXND1
15	BRWD1	55	PSG6
16	BTC	56	QSER1
17	CALCRL	57	RAB21
18	CASP2	58	RAPGEF2
19	CCDC132	59	RHOB
20	CCNA2	60	RPL18A
21	CD8A	61	SCUBE2
22	CDH4	62	SFTPA2
23	CLASP1	63	SLC38A1
24	CTBP2	64	SNAP29
25	CTGF	65	SNTB2
26	CTR9	66	STAT1
27	CUL5	67	SUCLG2
28	CYR61	68	TBC1D24
29	DPYSL5	69	TCEB3
30	EGFR	70	TM4SF18
31	EGR1	71	TOB1
32	EPC1	72	UACA
33	FBXO22	73	USP4
34	FNDC3B	74	VSTM2L
35	FOS	75	XRN1
36	GNAS	76	ZMYM2
37	GRIP1	77	ZNF217
38	GTF2A1		
39	HCK		
40	HLF		
